# Miocene Evolutionary History Shaped the Phylogeography of Willow Species (*Salix* L.) in the European Alps

**DOI:** 10.3390/biology15151299

**Published:** 2026-08-05

**Authors:** Loïc Pittet, Piotr Kosiński, Natascha D. Wagner, Elvira Hörandl

**Affiliations:** 1 Department of Systematics, Biodiversity, and Evolution of Plants (with Herbarium), University of Göttingen, 37073 Göttingen, Germany; loic.pittet@hotmail.com (L.P.); natascha.wagner@uni-goettingen.de (N.D.W.); 2 Georg-August University School of Science (GAUSS), University of Göttingen, 37073 Göttingen, Germany; 3 Faculty of Agriculture, Horticulture and Biotechnology, Poznań University of Life Sciences, 60-637 Poznań, Poland; kosinski@man.poznan.pl; 4 Institute of Dendrology, Polish Academy of Sciences, 62-035 Kórnik, Poland

**Keywords:** biogeography, demography, ecology, RAD sequencing, *Salix*, speciation

## Abstract

In the European Alps, plant species have persisted through major geological and climatic changes, leaving distinct genetic signatures of divergence. While these patterns are often attributed to Pleistocene glacial refugia, they may also reflect older, pre-Quaternary events. In mountain systems, lineage-specific ecological divergence, recurrent hybridization, and polyploidization can further complicate interpretations of genetic structure. Here, we combined RAD sequencing phylogenomic, demographic modelling, and ecological analyses to determine whether genetic variation in ten alpine willows (*Salix* spp.) reflects pre-glacial divergence or glacial dynamics, and to assess the role of extant environmental differentiation. Across all species, genetic data revealed consistent East–West structure. Demographic analyses indicate that lineage divergences predate Quaternary glaciations by several million years and are best explained by ancient founder events followed by periods of secondary contact. Ecological niche analyses identified differences in climatic and edaphic preferences, suggesting ecological divergence as an additional driver of lineage differentiation. Together, these findings demonstrate that the persistence of spatially differentiated yet incompletely diverged *Salix* lineages in the Alps was likely shaped by the combined effects of pre-Quaternary divergence, ecological selection, and recurrent gene flow, highlighting the importance of integrating ecological processes to understand evolutionary responses to climatic oscillations.

## Simple Summary

This study investigates how geological history, climate change, and ecological differences shaped genetic variation in alpine willow species across the European Alps. Using genomic, demographic, and ecological analyses, we found a consistent East–West genetic division across species. While Pleistocene glaciations influenced current distributions, most lineage divergences originated several million years before the Quaternary, likely driven by ancient range shifts. Ecological differences between lineages also contributed to their divergence. However, repeated periods of secondary contact and gene flow during glacial cycles prevented complete speciation. Our results highlight the combined roles of ancient evolutionary history, ecological adaptation, and gene flow in shaping biodiversity in the Alps.

## 1. Introduction

Mountain ecosystems are among the most dynamic and complex biogeographic regions, where environmental changes and evolutionary processes jointly shape species divergence and genetic diversity [1,2]. In the European Alps, plant species have persisted through geological transformations and dramatic climatic fluctuations, resulting in patterns of genetic variation that reflect both ancient divergence and recent postglacial recolonization [3,4]. The drivers and tempo of lineage diversification have been explored in several alpine herbaceous plant lineages such as *Androsace*, *Primula*, or *Phyteuma* [5,6]. However, these studies have revealed varying diversification forces. To gain a deeper understanding of how and when plant lineages diversified in mountain environments, further lineage-specific research is needed. In particular, subalpine and alpine shrubs remain largely understudied in this context.

The orogenic history of the European Alps spans over 100 million years, initiating already in the late Mesozoic and intensifying during the Cenozoic [7,8]. The uplift of mountain ranges has been widely recognized as a key driver of species diversification across various mountainous regions [9,10]. More recently, the gradual global cooling of temperatures (15 Mya) and the Pleistocene glaciations (starting c. 2.6 Mya) that repeatedly covered large parts of the Alps with an ice shield affected species distribution and diversification [11,12]. The impact of Pleistocene oscillations on diversification is, however, still debated. While harsh glacial conditions may have driven extinctions and suppressed diversification, repeated range fragmentation could have promoted species diversification [13].

The current genetic structure of plant species in the European Alps often shows remarkably similar spatial patterns, such as East–West differentiation or discontinuities aligned with major barriers [3,4,14,15,16]. These recurrent patterns have frequently been attributed to postglacial recolonization from glacial refugia and ecological factors [3,17]. However, ref. [14] demonstrated that shared genetic patterns may differ in temporal depth, with some differentiations dating back well before the Pleistocene. Early diversification may have been driven by ecological opportunity during and after mountain uplift, followed by a slowdown in diversification as ecological niches became filled [13,18]. Most studies have investigated recent postglacial dynamics, often overlooking older divergence events that might be key to understanding the full evolutionary history of alpine taxa. Moreover, plant species in mountain regions may show lineage-specific ecological divergence, as well as repeated instances of hybridization or polyploidization [17,19,20], complicating simple postglacial interpretations of genetic patterns [21,22,23]. These processes can generate lineage differentiation that is maintained despite range overlap, or mimic divergence created by isolation.

The genus *Salix* includes approximately 450 species of trees and shrubs, predominantly found throughout the Northern Hemisphere. Recent studies have provided new insights into the timing of species diversification and polyploidization within the genus [24,25,26]. The evolutionary history of willows has also been strongly influenced by hybridization and introgression, occurring both between sister species and among more distantly related lineages [19,20,27] (see [26] for a recent phylogeny of the genus). In the European Alps, there are approximately 33 species, which exhibit wide variation in geographic distribution, ecological preferences, and ploidy levels [28,29,30]. Willows, however, have a general preference for moist or wet soils and are mostly wind-pollinated. They are characteristic species for subalpine and alpine communities [31]. Willow species are perfect pioneer plants that occur among the first woody colonizers on newly available habitats [32,33]. While phylogeographic research in the Alps has largely focused on herbaceous species [3,13], subalpine and alpine shrubs like *Salix* remain understudied despite their ecological importance. As long-lived, woody perennials with long generation time and high dispersal potential, willows may have experienced evolutionary dynamics distinct from those of short-lived herbs. With its taxonomic diversity and diverse ecology, the genus *Salix* provides a valuable model for investigating how evolutionary processes, operating at different temporal scales, have shaped the genetic structure of subalpine and alpine plant lineages that we see today.

We include ten willow species (six shrubs and four dwarf shrubs) from the montane to alpine zone Table 1 [28]. These species represent a broad phylogenetic distribution across the subg. *Vetrix* (=*Chamaetia*/*Vetrix* clade) and do not form a monophyletic group within this clade [26,28]. Six of these species have previously been studied in the context of hybridization and polyploidization [19,20,27]. To capture the diversity of the genus in the Alps, we selected three polyploid species, two pairs of diploid sister species with vicariant distributions, and three additional diploid species spanning across the whole mountain range. Earlier studies found high genetic similarity within sister species pairs [19,27]; therefore, we treated them as single evolutionary units in most analyses, consistent with our approach for the other widespread species.

Here, we combine genomic data using Restriction Site Associated DNA (RAD) sequencing, demographic modelling, and ecological analyses to disentangle whether observed patterns in distributions of subalpine and alpine willows (*Salix* spp.) reflect ancient pre-glacial divergence or glacial dynamics, and to evaluate the role of ecological differentiation. We first characterize their current genetic structures. To go beyond these genetic snapshots, we estimate the timing of major intraspecific splits and use demographic models to reconstruct the processes behind them, including their origin and potential hybridizations during secondary contact. Finally, we assess ecological differences between intraspecific lineages. We did not analyze plastid regions because they are highly conserved and lack sufficient variation to resolve even species-level relationships, as demonstrated in the literature (e.g., using standard plastid barcoding markers [34] or complete plastomes [35]).

We test the following hypotheses: (1) all species or vicariant sister species pairs exhibit East–West patterns of genetic differentiation, similar to herbaceous plants [17]; (2) congruent spatial genetic patterns among species reflect shared historical drivers and tempos of evolution predating the Quaternary; (3) secondary contact hybridization following divergence (inter- and intraspecific) between eastern and western lineages is a shared feature across all species, and (4) ecological niche breadth and optimum of species differ within species between eastern and western part of the Alps, and among species. This comparative approach provides insights into the complexity of lineage diversification and temporal and spatial genetic structuring in the European Alps.

## 2. Materials and Methods

### 2.1. Sampling and Molecular Data Generation

Leaf material of the four species *S. appendiculata* (43 samples), *S. caesia* (23 samples), *S. helvetica* (39 samples), and *S. laggeri* (21 samples) was collected covering the complete Alpine species’ distributions. Additionally, RAD sequencing data of the six species *S. alpina* (82 samples), *S. breviserrata* (59 samples), *S. foetida* (56 samples), *S. retusa* (60 samples), *S. serpillifolia* (52 samples), and *S. waldsteiniana* (75 samples) were generated in previous studies (see Supplementary Information Data S1 for details). We also sampled two individuals per species from eight related taxa from the subg. *Vetrix* [26] to provide a broader phylogenetic context (Data S1). Each of the two individuals of the distantly related taxa *S. alba* and *S. triandra* [24] was used as an outgroup for phylogenetic analyses. *Salix alba* belongs to subg. *Salix*, while *S. triandra* is sister to subg. *Vetrix* [36]. Sampling of the ten focal species covered their entire distribution range within the Alps. For each population, between one and ten individuals were sampled, depending on local abundance. Because population sizes vary considerably in the subalpine and alpine habitats, sampling within populations was mostly random and could not follow a strict spatial scheme but avoided individuals that were too close to each other. Details on sampling, including locations and a summary of analyses performed for each individual, are provided in Data S1. Tetraploid (2*n* = 4×) ploidy for *S. laggeri* and *S. caesia* samples were confirmed by flow cytometry following the method of [20]; for ploidy levels of *S. retusa*, see [20].

Leaves were dried in silica gel, and herbarium voucher specimens were deposited in the herbarium of the University of Göttingen (GOET). The DNA was extracted from the dried leaf material using the DNeasy Plant Mini Kit (Qiagen, Hilden, Germany) following a modified manufacturer’s protocol [27]. After quality check, the DNA was sent to Floragenex Inc. (Beaverton, OR, USA) for library preparation following the protocol described in [37] and single-end RAD sequencing. The restriction enzyme PstI was used for digestion, and fragments between 300 bp and 500 bp were selected.

The program process_radtags.pl from Stacks v.2.65 [38] was used with default parameters to demultiplex raw reads, trim the reads to 86 bp and remove low-quality reads. The quality of the reads was checked using FastQC v.0.11.4 [39]. Because available *Salix* reference genomes were still scarce and distantly related to our focal taxa, de novo assemblies were performed. We used both Stacks and ipyrad v.0.9.52 [40], each optimized for different analytical aims. Stacks for intraspecific population genetic analyses (e.g., structure inference, demographic modelling) and ipyrad for multispecies datasets and phylogenetic contexts with higher divergence. Given that our study spans both population-level and interspecific analyses, employing both pipelines provide the most robust approach, maximizing marker recovery while minimizing methodological bias.

Stacks was used to build de novo loci for each species separately with the denovo_map.pl pipeline. Additionally, de novo loci were also built for the combined samples of each pair of sister species, *S. alpina* and *S. breviserrata*, and *S. foetida* and *S. waldsteiniana*. Combining sister species was based on previous studies reporting high genetic similarity between them [19,27], the absence of genetic structure within individual species, and the fact that vicariant sister species together exhibit an Alpine distribution similar to that of the widespread species. The maximum number of stacks at a single de novo locus was increased according to the ploidy of the species (Supplementary Information Table S1). The other parameters were optimized using a subset of samples and the r80 method [41,42], to maximize the number of polymorphic loci (Table S1).

In addition, ipyrad was used to run an assembly for a subset of 92 individuals. For each of the ten focal species, we selected three to four individuals from the STRUCTURE-defined genetic groups, prioritizing those with the least missing data. To provide a broader phylogenetic context, we included 16 individuals for eight related species. The clustering threshold was kept to 85%, as in previous phylogenetic studies on *Salix* subg. *Vetrix* [25,43,44]. Two alleles per site were allowed in the final consensus sequence, and the minimum number of samples sharing a locus was set to 69 (75%, see Table S1 for more details). The resulting sequences include all sites, both variant and invariant, and were used in the concatenated alignment for phylogenetic analyses.

### 2.2. Exploratory Analyses—Genetic Structure

STRUCTURE v.2.3.4 [45] was used to explore the patterns of genetic structure within species. The sister species *S. alpina* and *S. breviserrata*, and *S. foetida* and *S. waldsteiniana* showed no infraspecific genetic structure when analyzed individually (Supplementary Information Figure S1). We therefore reanalyzed each pair jointly, focusing on populations within the European Alps to identify potential substructure. The broader genetic structure of these pairs, including populations outside the Alps as well as the genetic structures of *S. retusa* and *S. serpillifolia* were characterized by [19]. *Salix retusa*, which is widespread across the European mountain system, exhibited a single genetic group across the Alps, possibly due to a stronger genetic differentiation at broader geographic scales [19,46]. In the present study, we reanalyzed the genetic structure of *S. retusa* with a focus on a potential substructure within the Alps.

For a detailed description of SNP filtering and data-processing workflow for the diploid and polyploid datasets, see Supplementary Information Section S1 and Table S1. STRUCTURE was run with the admixture model and correlated allele frequencies, but no other prior information [47]. Ten repetitions for each number of genetic clusters (K) were tested. Each run had 250,000 iterations as burn-in, followed by 500,000 MCMC iterations. Results were summarized using STRUCTUREHarvester v.0.7 [48]. The estimated log-likelihood and ∆K from the Evanno method were used to determine the optimal number of clusters [49]. CLUMPP v.1.1.2 was used to generate single Q matrices from the ten replicates [50].

Additionally, a principal component analysis (PCA) was performed on the same datasets using the R package adegenet v.2.1.10 [51]. Genetic structure analyses (STRUCTURE and PCA) consistently revealed two distinct clusters in each species, showing a recurrent East–West pattern. Accordingly, these western and eastern groups were treated as intraspecific phylogenetic units in all subsequent analyses.

### 2.3. Phylogenetic Relationships and Divergence Time

The phylogenetic relationships and relative age of the major intraspecific phylogenetic splits (STRUCTURE-defined genetic groups) were inferred in a single analysis including the ten focal species and eight related species, Maximum likelihood (ML) inference was conducted in RAxML v.8.2.4 [52] and divergence times were estimated with BEAST v.2.7.5 [53], both using the concatenated alignment of unlinked SNPs created with ipyrad (see Table S1). The RAxML analysis was performed with the GTR + Γ model of nucleotide substitution and 100 bootstrap replicates. For BEAST, we followed the settings in [25,26], applying an optimized relaxed clock approach, a GTR + Γ model of nucleotide substitution with four rate categories, and the Yule model for speciation. Time calibration used a uniform prior and a secondary calibration on the crown node derived from previous studies [24,25] together with the earliest reliable fossil from *Salix* subg. *Vetrix* [54], constraining its age between 23 and 51 Ma. STRUCTURE-defined groups were constrained to be monophyletic. Two independent MCMC analyses were run with 10 million generations, and the outputs were investigated with Tracer v.1.7.2 to assess convergence [55]. We combined the outputs with LogCombiner from the BEAST package, discarding 25% of the logs as burn-in. A maximum clade credibility tree with common ancestor heights was generated with TreeAnnotator v.1.10 [56].

### 2.4. Demographic Modelling

The diffusion approximation method of dadi was used to analyze two-dimensional joint site frequency spectra (JSFS) and test demographic scenarios [57]. Groups were defined from STRUCTURE results at K = 2, assigning individuals with <10% admixture to their respective group. For a detailed description of SNP filtering, see Supplementary Information Section S1 and Table S1. Folded JSFS were inferred from VCF files using the software easySFS (https://github.com/isaacovercast/easySFS; accessed on 3 October 2025), which performs down-projection of groups to account for missing data and maximize the number of segregating sites (for number of individuals included per species and projected group sizes, see Supplementary Information Table S2).

The 2D optimization routine implemented in dadi_pipeline v3.1.7 was used to fit predefined models [58]. All models were optimized over four consecutive rounds with 60, 80, 80, and 100 replicates, respectively. Each round was seeded with parameter estimates from the best-scoring replicate of the previous round. The best performing model was selected based on the Akaike information criterion and log-likelihood. Specifically, two sets of demographic models were used. The first set tests the origin of the defined genetic groups and allows the differentiation between vicariance and founder event (for all alternative demographic scenarios tested, see Supplementary Information Figure S2, [59,60]). These models are sensitive to directionality and account for the initial size of the founding population. For each case, we tested all models in both directions. The second set tests the diversification between the defined genetic groups and allows for the differentiation between isolation periods, periods of gene flow and secondary contact (for all alternative demographic scenarios tested, see Supplementary Information Figure S3, [58]). To assess whether the selected demographic model adequately captured the observed JSFS and to estimate divergence times between the intraspecific phylogenetic groups, we conducted goodness-of-fit tests following [61]. Details are provided in Supplementary Information Section S2. Divergence times between the intraspecific phylogenetic groups estimated from the origin tests were subsequently transformed into absolute time using a 30-year generation time which can be regarded as typical for alpine willows (see also Section S3) [62]. In the absence of reliable data for close relatives, we used the mean mutation rate of *Arabidopsis thaliana* 7 × 10^−9^ [63]. If the distribution of the divergence times was not Gaussian or showed broad confidence intervals, we concluded that the data contain limited information to reliably estimate the parameter [64].

### 2.5. Ecological Variables

All environmental predictors were aggregated (by geographical average) to a 250 m spatial resolution and projected to the standard Lambert Azimuthal Equal Area projection for Europe (ETRS89/LAEA Europe; EPSG:3035; EPSG Geodetic Parameter Dataset).

Soil and topographic variables were obtained from the Global Lithological Map [65], SoilGrids [66], and the GMES RDA project (EU-DEM, www.eea.europa.eu/data-and-maps/data/, accessed on 1 November 2024). GLiM soil lithology was used to generate a bedrock-type layer following [67]. SoilGrids variables describe topsoil properties that are ecologically relevant for plant species (for details, see Supplementary Information Table S3). Climatic conditions over the period 1979–2013 were obtained from the Chelsa Climate database https://chelsa-climate.org (accessed on 1 November 2024) [68,69]. We retained twelve biologically relevant variables with weak correlation (Pearson’s |r| < 0.7; Supplementary Information Figure S4) and variance inflation factor VIF < 5. All variables considered and kept are listed in Table S3.

### 2.6. Ecological Niche Comparison

**Figure 1:**
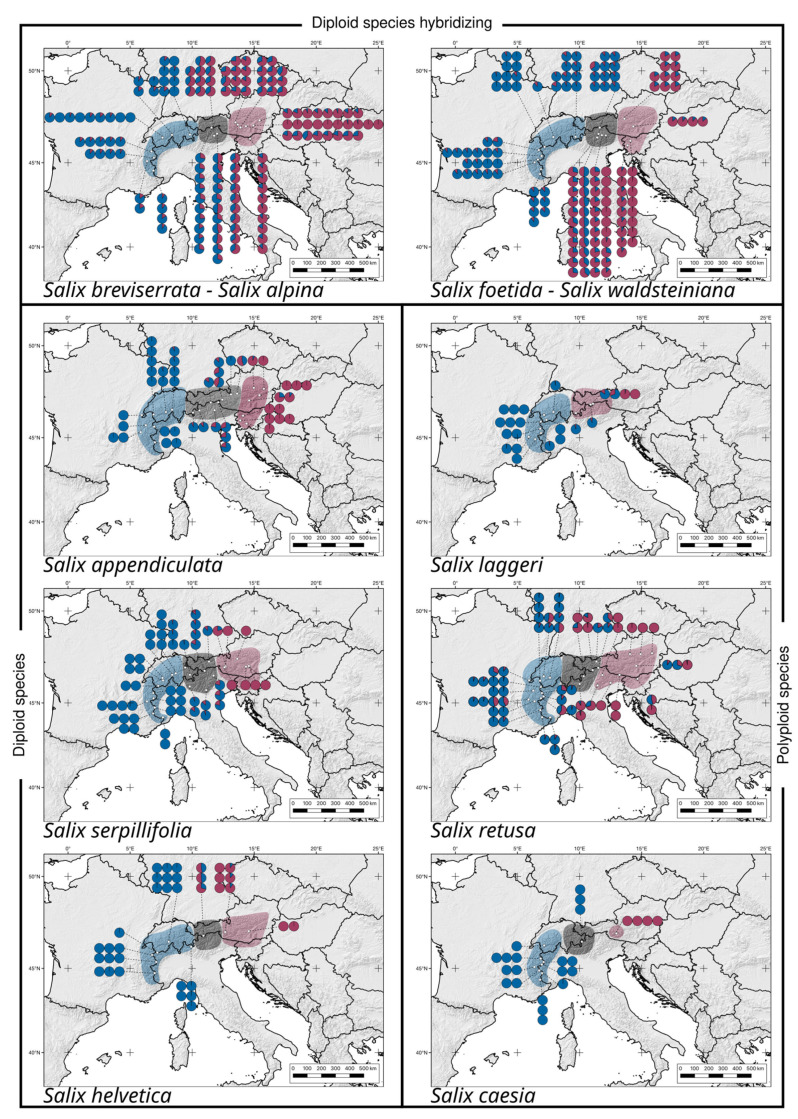
Genetic structure of alpine willows across the European Alps. Pie charts represent the optimal STRUCTURE results for K = 2; multiple pie charts at a single location indicate that more than one individual was sampled at that site. The coloured background shapes (blue = western genetic group, red = eastern genetic group, grey = admixed group) illustrate the approximate areas associated with the three STRUCTURE-defined genetic groups. These areas, assigned based on the proximity of occurrence records to sampling points with known genetic affiliation, as described in the Methods, were subsequently used to assess ecological differentiation between genetic groups within species.

We compared the environmental niches of lineages defined by the genetic structure, emphasizing broad-scale patterns in their environmental separation. Based on the STRUCTURE results, we defined three occurrence areas for niche comparison (represented in Figure 1): one corresponding to the western genetic group, one to the eastern genetic group, and a third representing the central area where admixed individuals were found. Precise occurrences of all species across their Alpine distribution were gathered from the European Vegetation Archive EVA database [70], Infoflora (https://www.infoflora.ch/, accessed on 28 April 2022), GBIF database (https://www.gbif.org/, see Supplementary Information Section S4 for DOI and accession dates), Silene Expert (https://silene.eu, accessed on 14 June 2021), Biodiv’AURA Expert (https://donnees.biodiversite-auvergne-rhone-alpes.fr/, accessed on 14 June 2021), and our collections. Strong spatial and resolution filtering was applied to the compiled data (for more details on the procedure, see Supplementary Information Section S5). Our final observational data set comprised 4837 occurrences. These filtered occurrence records were manually assigned to one of the three areas (STRUCTURE-defined genetic group) based on their proximity to individuals with known genetic affiliation (Supplementary Information Table S4).

Direct comparison of niches required species observations and the definition of a background area, including suitable habitats. We defined a 5 km buffer zone around the occurrences of each species and sampled 1000 observations per species to generate the background area. Niches were compared following the method described by [71] and implemented in the R package ecospat v.4.1.2 [72]. We projected species occurrences onto a two-dimensional grid of 100 × 100 cells derived from principal component analysis (PCA). Additionally, kernel smoothing was applied to estimate the density of occurrences of each species in the PCA without overemphasizing outliers or gaps in the data. Using the multivariate space, we compared niche overlap using Schoener’s *D* metric with the correction of the occurrence densities of each species by the prevalence of the environments in their range [73]. Niche divergence and niche conservatism were tested using the niche equivalency and niche similarity tests, respectively [71,74]. Niche optimum and niche breadth along the PCA axes were calculated by randomly subsampling 100 cells of the gridded space. The resampling was repeated 1000 times, and niche optimum was computed as the mean of the sampled scores. Niche breadth was calculated as the difference between the 97.5% and 2.5% quantiles of the sampled scores.

## 3. Results

### 3.1. Exploratory Analyses—Genetic Structure

For all six single species and the two sister species pairs included in this study, STRUCTURE analyses consistently identified K = 2 as the optimal number of genetic groups (Supplementary Information Figure S5). A recurrent East–West pattern was observed in each case (Figure 1). One group (blue) corresponds to the Western Alps, representing either a distinct genetic cluster in single-species analyses or the western taxon in sister species comparisons; the other group (red) corresponds to the central and eastern part of the Alps. Individuals showing genetic admixture were detected in all species except *S. caesia*. They were predominantly located in the intermediate region, particularly from eastern Switzerland to western Austria (North Tyrol) and northeastern Italy (South Tyrol).

The genetic patterns inferred from STRUCTURE were further supported by the PCA analyses (Supplementary Information Figure S6). In all cases, the first principal component clearly distinguished individuals assigned to different genetic groups. Most of the admixed individuals occupied intermediate positions along the first axis. The second principal component further differentiated individuals within the genetic groups. In the two sisters-species *S. alpina*-*S. breviserrata* and *S. foetida*-*S. waldsteiniana*, the eastern group appeared to exhibit higher genetic diversity compared to the western cluster. The opposite was observed in *S. appendiculata*, *S. serpillifolia*, *S. laggeri*, and *S. caesia.* In *S. helvetica* and *S. retusa*, both genetic groups exhibited substructure along the second principal component.

### 3.2. Divergence Time Estimation

**Figure 2:**
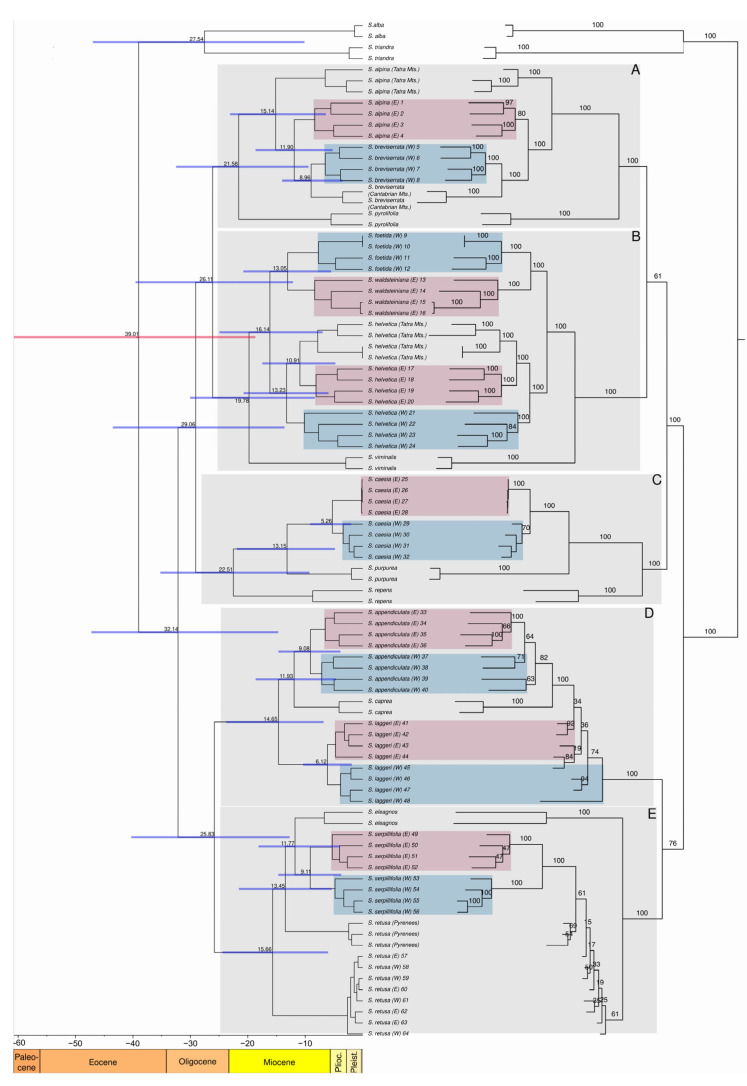
BEAST divergence estimation tree (left) and RAxML phylogenetic tree (right) based on 17,348 loci (315,876 SNPs) and 92 samples representing intraspecific lineages and related species. The trees were rooted with two outgroup species (*Salix alba* and *Salix triandra*). The ten focal species (Clade A–E) belong to *Salix* subg. *Vetrix* and are grouped into five major clades. Lineages are colour-coded according to their genetic structure (Figure 1), with western and eastern genetic groups indicated by (W) and (E), respectively. Numbers refer to individual IDs in the column “BEAST” of the Supplementary Information Data S1, which includes details on sampling location. (Left) Calibration point for the divergence time estimation is indicated by the red confidence interval. Blue bars represent the confidence intervals at specific nodes, with the ages at the nodes being the mean. (Right) Bootstrap support for the phylogenetic relationship is indicated on top of the branches.

The ML tree and BEAST results are shown in Figure 2. Both analyses resulted in almost similar topologies in most nodes. The outgroups *S. alba* and *S. triandra* were well separated from the subg. *Vetrix* (BS 100). The divergence time between the outgroup and the ingroup was estimated at 39 Mya. The subg. *Vetrix* was further divided into five fully supported clades (A–E). Clade A included the first sister species pair, *S. breviserrata* and *S. alpina.* The second sister species pair, *S. foetida* and *S. waldsteiniana*, was grouped together with *S. helvetica* in clade B. Clade C represented *S. caesia.* Samples of the tetraploid *S. laggeri* grouped with *S. appendiculata* and *S. caprea* in Clade D. Finally, *S. retusa* and *S. serpillifolia* formed the clade E. In the different clades, most of the branches were fully supported. Except for the polyploid species *S. laggeri* and *S. retusa*, the RAxML tree recovered well-supported, monophyletic groups corresponding to the STRUCTURE-inferred clusters (Figure 2). The BEAST result indicated that the estimated phylogenetic splits between western and eastern groups within species or between sister species varied from 5.26 to 13.23 Mya (except for *S. retusa*, where the divergence was not well resolved). For these splits, the lower boundaries of the 95% Highest Posterior Density (HPD) were found in polyploid species (1.61 Mya and 1.83 Mya for *S. caesia* and *S. laggeri*, respectively).

### 3.3. Demographic Modelling—Origin of Genetic Groups

**Figure 3:**
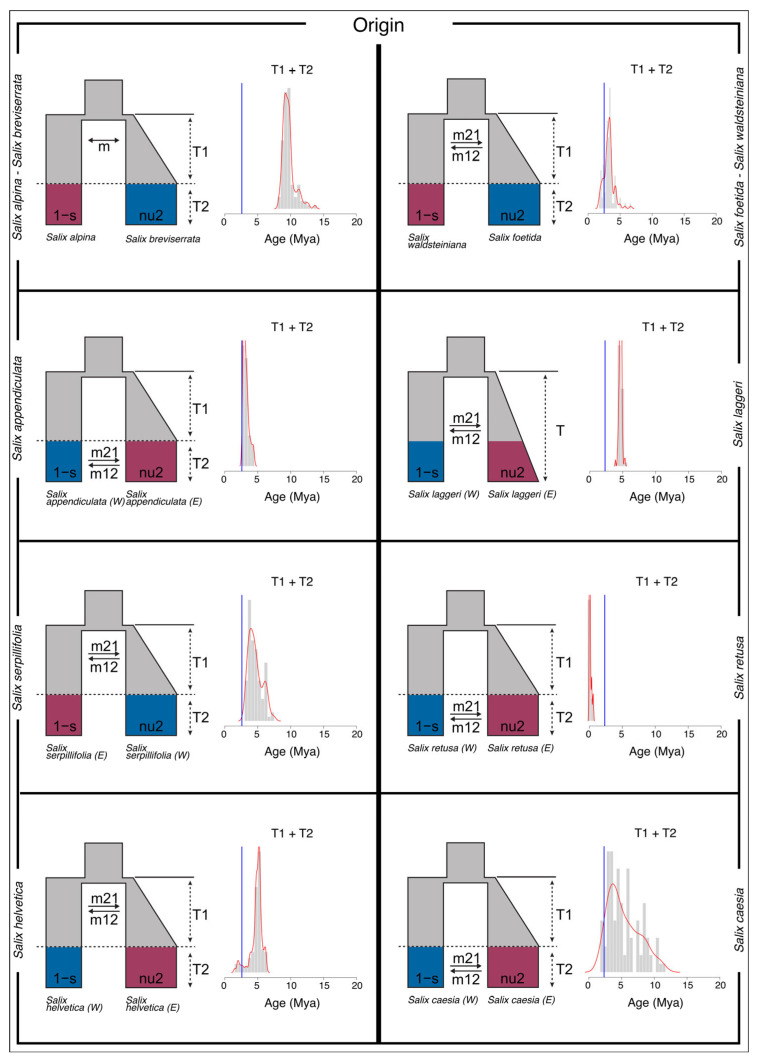
Best performing demographic models for the origin of lineages. For each species, the schematic visualization of the best model (left) and the distribution of the estimated divergence time (right) are displayed. The blue line indicates the beginning of the Pleistocene climate oscillations (2.6 Mya), the gray bars represent the frequency distribution of the estimated divergence times, and the red line indicated the corresponding trend. Abbreviations: nu2 = effective population size of the diverged population; m = symmetric migration; m12/m21 = migration from population 1 to population 2, and from population 2 to population 1, respectively; T1/T2/T3 = time period 1, 2, 3, respectively; s = founding fraction for the origin models. For more details, see [58,59]. Parameters’ estimates can be found in Table 2. The empirical and modeled JSFS, residuals between the empirical and modeled JSFS, and the empirical log-likelihood and its distribution from the goodness-of-fit test (100 replicates) are shown in Supplementary Information Figure S7.

In all comparisons, models representing founder events fitted better to the data than vicariance models (Table 2, Figure 3, Supplementary Information Tables S5–S12). In the case of *S. alpina*-*S. breviserrata*, *S. foetida*-*S. waldsteiniana*, and *S. helvetica*, the eastern group represented the ancestral (source) population. In all other cases, the ancestral population was represented by the western group. For *S. laggeri*, the best-fitting model indicates a split from the ancestral population followed by exponential growth of the derived population, with ongoing asymmetric gene flow. In the cases of *S. retusa* and *S. caesia*, the models support a split with subsequent exponential growth of the derived population without gene flow, followed by a period of constant population size during which asymmetric gene flow resumed. For all remaining species, the best models suggest a split followed by exponential growth and gene flow, transitioning into a phase of isolation with constant population sizes and no further migration. In all cases except for *S. retusa*, gene flow was strongly asymmetric, occurring primarily from the source to the derived population (Table 2). The founding fractions of the derived populations were consistently small, ranging from 3% to 15% (Table 2).

For all species except *S. caesia*, the empirical log-likelihood values aligned well with the estimated distributions, the residuals showed no strong systematic deviations, and the inferred timing parameters were associated with comparatively narrow distributions, all pointing to a relatively good model fit (Figure S7). Notably, the estimated origins of the intraspecific groups predated the onset of the Pleistocene climatic oscillations (2.6 Mya) in all cases except for *S. retusa* (Figure 3). Halving generation times would still date the splits of *S. alpina-S. breviserrata*, *S. laggeri*, *S. serpillifolia*, and *S. helvetica* before the Pleistocene. The highly polyploid species *S. retusa* exhibited more pronounced residual structure, which may reflect biological complexity that is not fully captured by the demographic model (Figure S7) and therefore calls for a cautious interpretation of the results. In the case of *S. caesia*, the simulated and empirical JSFSs showed discrepancies, suggesting that the model did not fully capture the underlying demographic signal (Supplementary Information Figure S7). The goodness-of-fit assessment showed that the AIC-selected model failed to recover reliable parameter estimates, as indicated by the broad distribution of inferred timing parameters and expected log-likelihoods.

### 3.4. Demographic Modelling—Diversification Mode of the Genetic Groups

**Figure 4:**
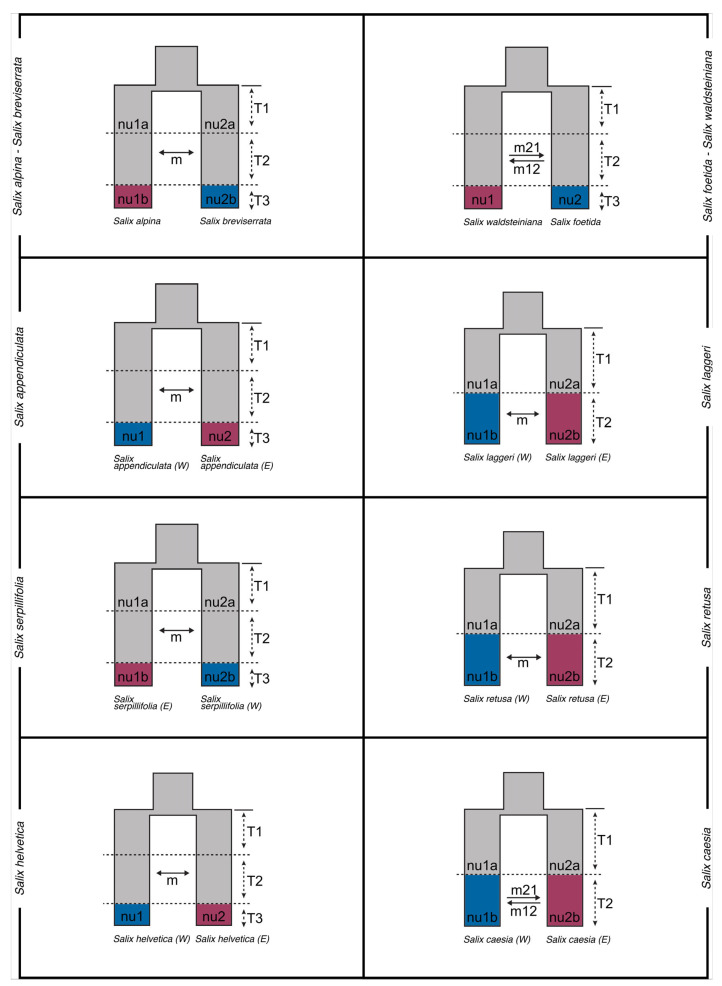
Best performing demographic models for the diversification of lineages. For each species, the schematic visualization of the best model is displayed. Abbreviations: nu1 = effective population size of the source population before (a) and after (b) the expansion; nu2 = effective population size of the diverged population; m = symmetric migration; m12/m21 = migration from population 1 to population 2, and from population 2 to population 1, respectively; T1/T2/T3 = time period 1, 2, 3, respectively. For more details, see [58,59]. Parameters’ estimates can be found in Table 2. The empirical and modeled JSFS, residuals between the empirical and modeled JSFS, and the empirical log-likelihood and its distribution from the goodness-of-fit test (100 replicates) are shown in Supplementary Information Figure S8.

The results showed that the models including secondary contact following an initial period of isolation provided the best fit to the data (Table 2, Figure 4, Supplementary Information Tables S13–S20). For the polyploid species *S. caesia*, *S. laggeri*, and *S. retusa*, the best-fitting models consisted of only two different epochs, with no subsequent isolation phase. In contrast, the remaining species’ genetic patterns were best explained by models that included a third epoch of isolation after secondary contact. Gene flow was consistently high during the secondary contact phase. The final isolation phase was generally much shorter than the preceding periods (Table 2).

For this second set of models, the empirical log-likelihood mostly fell within the posterior distributions generated from the simulated datasets (Supplementary Information Figure S8). However, these posterior distributions were substantially broader, indicating that the model provided a weaker overall fit compared with the first set of models. In *S. caesia*, the goodness-of-fit results showed that the model failed to reproduce the empirical data adequately (Figure S8). Because this set of models exhibited a generally poorer fit than the previous one, we chose to convert demographic parameters into biological units only for the first set.

### 3.5. Ecological Niche Differentiation

**Figure 5:**
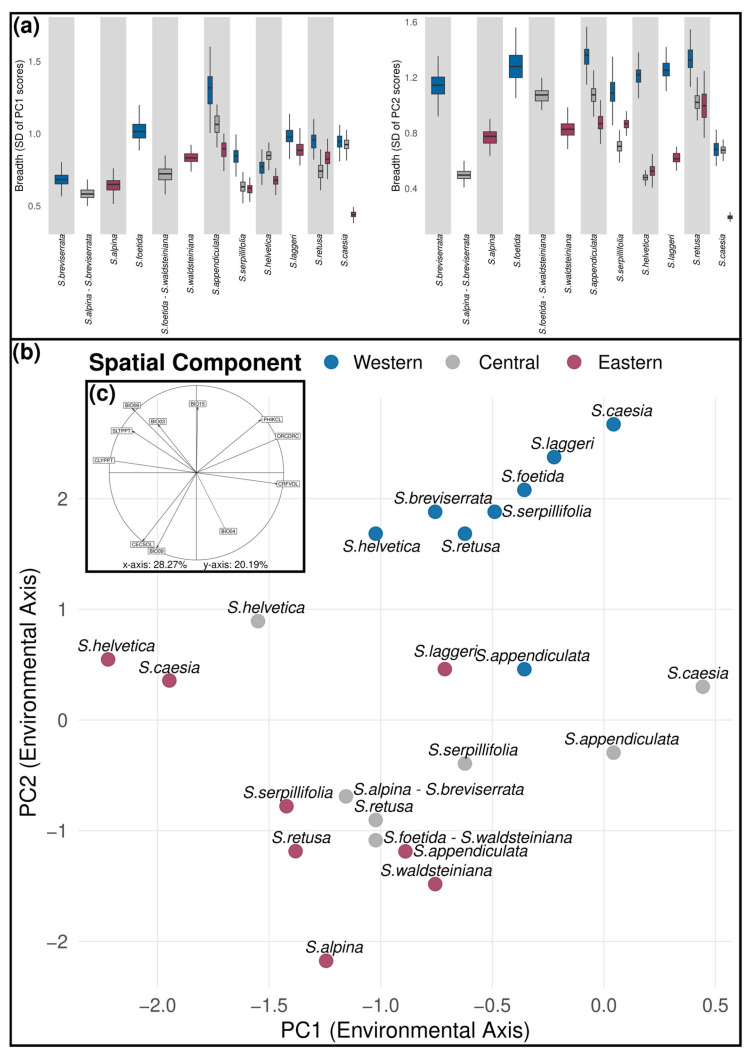
Ecological comparison within and between species. (**a**) Niche breadth along first (left) and second (right) axis of the two-dimensional space obtained by principal component analysis from the soil and bioclimatic variables. Boxplots are centered at the mean, quartiles and whiskers indicate standard deviation and total variation, respectively. (**b**) Niche optima in the two-dimensional space obtained by principal component analysis from the soil and bioclimatic variables. Colors correspond to the regions defined by genetic groups (blue for western genetic group, red for eastern genetic group, grey for the admixed group in the central part). (**c**) Contribution of soil and bioclimatic variables to the axes of the two-dimensional space obtained by principal component analysis. Abbreviations: BIO03 = Isothermality, BIO04 = Temperature seasonality, BIO08 = Mean temperature of wettest quarter, BIO09 = Mean temperature of driest quarter, BIO15 = Precipitation seasonality, CECSOL = Soil Cation exchange capacity, ORCDRC = Soil organic carbon density, CRFVOL = Soil coarse fragments, PHIKCL = Soil pH in KCL, CLYPPT = Soil clay content, SLTPPT = Soil silt content.

The first two PCA components explained 48.46% of the total variation in the environment. PC1 mostly corresponded to soil variables, while PC2 was more correlated to climate variables (Figure 5a). The niche comparisons showed different levels of overlap, with Schoener’s D ranging from 7.7% to 54.1% (Table 3, Supplementary Information Figure S9). Only the niches of *S. alpina*-*S. breviserrata* were significantly less equivalent than expected by chance. In contrast, the niches of *S. appendiculata*, *S. serpillifolia*, and *S. helvetica* were more similar than expected by chance, indicating niche conservatism. Niche optima differed significantly along both PC1 and PC2 in all pairwise comparisons. A plot of niche optima across species reveals a clear separation between western, central, and eastern Alpine regions, with most of the variation occurring along PC2, which primarily reflects climatic gradients. Additionally, eastern niches were significantly narrower than western niches along both PCA axes, except for *S. caesia*, which did not show a significant difference in niche breadth (Table 3, Figure 5).

## 4. Discussion

### 4.1. Evolutionary History and Origin

Species diversification in mountain environments has long been associated with the geological uplift of mountain ranges, which creates complex topography and heterogeneous habitats that promote isolation and divergence [9,10]. In the case of the European Alps, both the orogeny and subsequent climatic changes have shaped evolutionary trajectories [11,75]. The existence of multiple glacial refugia around the periphery of the Alps suggests that recent glacial periods influenced lineage diversification [76], either by promoting allopatric fragmentation or by enabling secondary contact during interglacial periods [13,77,78].

Our genetic results consistently reveal an East–West differentiation within species and between sister-species pairs (Figure 1 and Figure S6), corresponding to one of the most prominent phylogeographic break zones in the Alps, the Brenner line [3,16,79,80]. Recognized since the 19th century [81] and later emphasized by Merxmüller [82] as delimiting a transitional floristic zone and potential contact region, the Brenner line has repeatedly been shown to separate major centers of species diversity and endemism [4,83]. In particular, Thiel-Egenter et al. [84] identified two major discontinuities in floristic composition and intraspecific genetic structure, one of which closely corresponds to the break recovered in our study. The intraspecific lineage patterns recovered in our willow species corroborate the importance of this barrier, although reflecting more a zone spanning over 200–300 km rather than a “line” [19]. Despite their high dispersal capacity and differing soil preferences, all species exhibit genetic structures that mirror the Brenner zone, suggesting that additional ecological and historical processes beyond simple dispersal limitation or edaphic niche differentiation have shaped East–West differentiation in the Alps.

Intraspecific lineage divergence, the formation of areas of endemism, and the emergence of unique diversity in isolated mountain ranges of the European Alps are often attributed to effects of Pleistocene climate oscillations [17,85,86]. While glacial refugia and post-glacial recolonization undoubtedly influenced the current spatial genetic patterns [87], it would be reductive to attribute current genetic differentiation solely to these recent climatic events. Instead, our results, supported by a time-calibrated phylogeographic analysis (Figure 2) indicate that most East–West divergences predate the Quaternary glaciations, with estimated splits between major genetic lineages ranging from means of 5.26 to 13.23 million years ago. Divergence time analyses are inherently challenging, as estimates depend strongly on calibration points, prior settings, and methodological approaches, making precise inference of evolutionary timelines difficult [88]. Therefore, the range of divergence times should be interpreted with caution. Nevertheless, with the exception of *S. retusa*, the 95% highest posterior density intervals of lineage divergence estimates fall within the Miocene, supporting the hypothesis that Quaternary climatic oscillations were not the primary drivers of lineage diversification. The time-calibrated tree topology was consistent with the Maximum Likelihood phylogeny, which received high bootstrap support for the main clades (Figure 2). Our divergence time estimates are consistent with the increase in speciation rate reported for the *Chamaetia*/*Vetrix* clade [25] and support its diversification driven by migration and dispersal across Eurasia, likely facilitated by mountain uplift during the Miocene as concluded in Marinček et al. [26]. With a few notable exceptions, our estimates are congruent with previous phylogenetic studies that sampled most taxa within the clade [24,25], including all sister taxa and the focal species investigated here. The largest discrepancies involved the hybridizing species pairs *S. alpina*-*S. breviserrata* and *S. foetida*-*S. waldsteiniana*. Previous divergence estimates were likely underestimated due to introgressed samples from the secondary contact zones, as gene flow between sister species biases divergence times downward [89]. These results emphasize the importance of accounting for genetic structure and introgression when estimating divergence times.

These deep divergences are further supported by the conversion of demographic parameters into biological units derived from the dadi modelling (Figure 3). Under the assumed generation time of 30 years and a mutation rate estimated from *Arabidopsis thaliana*, all lineage divergences except that of *S. retusa* predate the Quaternary, indicating that Pleistocene climatic oscillations were unlikely to have been the primary drivers of diversification. We acknowledge that both the generation time and mutation rate are uncertain, as species-specific estimates are currently unavailable for alpine species of *Salix*. Consequently, the absolute timing of demographic events should be interpreted with caution. Nevertheless, although alternative values would shift the estimated divergence times, the concordance between the demographic modelling and the independent BEAST dating provides consistent support for a pre-Quaternary origin of most lineages. While substantially shorter generation times would reduce the estimated divergence dates, they would still indicate pre-glacial divergence for four lineages but would become increasingly inconsistent with the independent age estimates inferred from BEAST. Only *S. retusa* exhibited a more recent split, consistent in both the BEAST analysis and the demographic modelling. However, these results should be interpreted with additional caution, as they may reflect methodological artefacts related to its high ploidy level, the bigger genome size and consequently higher mutation rate [90] and the current lack of bioinformatic tools capable of fully accounting for such genomic complexity.

Such pre-glacial diversifications have been found in the European Alps in other genera as well in *Primula* and *Androsace* [5], in *Gentiana* [91], in *Saxifraga* subsect. *Arachnoideae* [92], in *Ranunculus* sect. *Aconitifolii* [93,94]. Moreover, they have been documented frequently in other mountain systems worldwide [77,95,96,97]. These findings strongly support the hypothesis that much of the genetic diversification originated already after the last major uplift of the Alps (c. 30 Mya), likely influenced by newly emerging ecological gradients such as soil heterogeneity, microclimatic niches, and gradual cooling of temperatures [13,98]. Geological processes have further determined the large-scale distribution of siliceous and calcareous bedrock in the Alps, thereby shaping soil properties that persist to the present day.

Allopatric speciation has long been considered the predominant mode of diversification in mountain environments [99]. In our study, demographic model testing consistently indicated that the observed East–West genetic patterns are best explained by ancient (pre-glacial) founder events (Table 2, Figure 3). Therefore, speciation appeared to be peripatric rather than by vicariance, plausibly driven by the island-like distribution of habitats at high elevations [1]. The inferred directionality of these events, however, varied among species and potentially reflects differences in ancestral distributions or ecological preferences. The topology of the phylogenetic trees further supported the directionality inferred from demographic simulations, as all source populations (Table 2) consistently appeared older than the derived ones (Figure 2).

For the two sister species pairs, *S. alpina*-*S. breviserrata*, and *S. foetida*-*S. waldsteiniana*, as well as for the species *S. serpillifolia*, the eastern populations were consistently identified as the source of westward founder events. This pattern may suggest a colonization history originating from Central Asia, as previously proposed for the two sister species pairs [19], consistent with results from other alpine plant groups. The species-rich genus *Gentiana* L. shows strong affinities between the European Alps and Asian mountain ranges, and an Asian origin of European mountain taxa has been confirmed [79,100]. Connections between the Eastern Alps and the Carpathians have recently been identified for multiple subalpine and alpine species as well [16]. In contrast, for *S. retusa*, our results align with a previous hypothesis suggesting a Pyrenean origin, followed by eastward colonization into the Alps [20]. For *S. helvetica*, demographic models identified western populations as the source and further eastward migration. Eastward colonization from the Pyrenees to the Balkan Peninsula and intra-Alpine genetic structure has been observed in *Anthyllis montana* L. [8,101]. The two tetraploid species, *S. laggeri* and *S. caesia*, are endemic to the European Alps, and their origins are likely more complex, potentially having arisen via allopolyploidy from European progenitor species [44] in western Alpine regions. Although *S. caesia* has also been reported from Central Asia, for instance in Mongolia [102], a preliminary phylogenetic analysis suggested that these extra-European occurrences are genetically unrelated. Morphological assessments of Mongolian herbarium specimens further confirm clear differences between European and Asian *S. caesia*. However, a formal taxonomic revision is beyond the scope of this study.

Recent molecular phylogenetic studies increasingly challenge the palaeobotanical view that plant speciation was rare during the Quaternary, instead showing that climatic oscillations often drove diversification, particularly in mountainous regions [78]. However, our results align more closely with the “evolutionary stasis” hypothesis proposed by Bennett [103], which argues that many plant species underwent limited evolutionary change despite drastic environmental fluctuations. In our case, both species identity and intraspecific differentiation have remained largely stable since the Miocene, supporting the idea that the Quaternary primarily influenced population substructure rather than species diversification [14]. Nonetheless, repeated glacial-interglacial cycles likely facilitated episodes of secondary contact and introgression, possibly slowing the divergence of otherwise ecologically distinct lineages.

### 4.2. Post-Origin Diversification and Colonization Processes

In contrast to many alpine taxa, where allopatric diversification frequently led to speciation [78], most willow lineages have not been recognized as distinct species despite deep divergence times. Only the most morphologically different lineages are classified as sister species, although their divergence estimates are comparable to other intraspecific lineages (Figure 2 and Figure 3). Our demographic models testing diversification consistently identified phases of secondary contact following initial divergence (Table 2, Figure 4). In all diploid species, these periods of gene flow were framed by periods of isolation. Combined with the divergence time estimates, the results suggest a scenario in which pre-Quaternary mountain uplift and climatic cooling initially drove founder events and lineage divergence. Unlike many plant species [78], the following long interglacial periods of the Pleistocene likely enabled recolonization and intermittent gene flow between diverging lineages in *Salix*, preventing complete allopatric speciation [104]. Gene flow in willows is fostered by long life spans, and by wind-dispersed pollen and seeds [32,105]. They are among the first colonizers on glacier forefields, suggesting a fast recolonization rate [106,107]. Similarly, a slowdown in species diversification of the Primulaceae family during the Pleistocene has been hypothesized to result from migration and reconnection between isolated populations due to the climate oscillations [13]. By contrast, in our case, the relatively short time since the Last Glacial Maximum has not yet permitted substantial gene flow or introgression between some of the most geographically distant diploid individuals (such as those showing no admixture in the STRUCTURE analyses and used for demographic modelling).

The secondary phase of isolation, however, was not observed in the polyploid species. In these cases, discrepancies between the empirical and modelled SFS were more pronounced, and the empirical log-likelihoods in comparison with the distributions obtained in the goodness-of-fit tests indicated poorer overall fits of the demographic models. These results should therefore be interpreted with caution. The reduced model performance may reflect the species’ higher ploidy level, which is not accounted for in standard demographic inference frameworks. In *S. retusa*, this pattern may however also reflect an increased adaptive and ecological potential in polyploids, enabling them to maintain connectivity between lineages during the last glaciations [108,109]. Additionally, its potentially greater colonization capacity may have enabled *S. retusa* to recolonize the Alps more rapidly after the last glaciation, promoting earlier and more widespread introgression across the range [110]. This hypothesis is less plausible for *S. caesia* and *S. laggeri*, because both species have scattered, local distributions and are much less abundant than their diploid relatives [44]. For both species, recent anthropogenic loss of habitats (vulnerable/endangered in Switzerland and Austria; see Infoflora, https://www.infoflora.ch/ (accessed on 3 March 2026) and Schratt-Ehrendorfer et al. [111], respectively; see Data S1 for details) reduced our genetic sampling and created more pronounced imbalance in sample sizes between the two genetic groups, which could have affected demographic modelling. Nonetheless, across all species, the genetic structure within the Alpine range reveals ongoing admixture during the postglacial period, highlighting a dynamic and continuous process of recolonization and hybridization. This interplay between deep-time divergence and recent secondary contact provides a more nuanced understanding of subalpine and alpine lineage diversification.

### 4.3. The Impact of Extant Ecological Differentiation

The deep-time genetic divergence raises questions about the role of ecological differentiation in maintaining lineage separation over millions of years. In cases of incomplete speciation, as seen in our data, isolation driven solely by genetic drift would likely be eroded by intermittent gene flow [112]. The persistent genetic distinction observed between eastern and western lineages, despite episodes of secondary contact, suggests that ecological factors at large scale may contribute to some level of reproductive isolation. Indeed, our ecological niche comparisons revealed differences in current climatic and edaphic preferences among divergent lineages (Figure 5, Table 3). This result suggests that abiotic conditions contributed to lineage diversification [113].

Major differences between lineages were found along four ecological variables (Figure 5). Western lineages tend to prefer soils with higher pH and lower cation exchange capacity, whereas eastern lineages are associated with warmer climates and stronger seasonal variability. For several species, niche overlap between their eastern and western genetic lineages was notably low (see Table 3). These results are consistent with previous studies showing that adaptation to distinct environmental conditions can reinforce genetic differentiation in alpine plants or complement geographic divergence [17,114].

Notably, eastern lineages consistently exhibited narrower niche breadths compared to their western counterparts, a pattern that correlates with the broader geographic distribution of western lineages. This asymmetry may reflect environmental factors that favored postglacial recolonization in the Western Alps, including a greater diversity of microhabitats due to a fine-scale mosaic of bedrock types, the presence of higher mountains offering more extensive alpine areas with diverse habitats, and the wetter climatic conditions favorable to *Salix* [30]. Interestingly, the polyploid species did not consistently exhibit broader ecological niches (Figure 5a). Our results, however, align with previous findings showing that the polyploid *S. retusa* has a wider niche than its diploid relative *S. serpillifolia*. Among all species, the widespread diploid *S. appendiculata* showed the broadest niche breadth, which is expected given its distribution across a wide range of elevation from montane to subalpine zones. While polyploid species did not have broader niches overall, they tended to occupy more extreme ecological niche optima compared to the diploids (Figure 5b).

Together, these findings suggest that ecological divergence has contributed to maintaining genetic structure across the Alps and shaping current distributions. However, several limitations must be considered, as we have observed just large-scale correlations without confirmation by experimental and physiological studies. Particularly, regarding the soil variables. SoilGrids provides modeled, interpolated soil properties at relatively coarse spatial resolution, which may not capture the fine-scale edaphic heterogeneity characteristic of alpine and subalpine environments. In steep mountain systems, soil depth, moisture, and substrate composition can vary substantially over short distances, and such microhabitat variation is unlikely to be fully represented in gridded products. Therefore, soil-related niche differences inferred here should be interpreted cautiously and as broad-scale trends rather than precise local ecological differentiation. Our soil characteristics, however, fit to large-scale characteristics as reported, e.g., in [30,32].

Fine-scale differences between individual localities would require higher-resolution field-based soil data. Notably, similar East–West patterns have been observed in detailed analyses of sister species pairs using co-occurrence data and Landolt indicator values suitable for alpine and subalpine environments. These studies show that sister species have similar but not identical edaphic niches, with the western species consistently exhibiting a broader niche, supporting the broad trends observed in our study [19].

The interaction between ancient divergence, glacial connectivity, and ecological differentiation appears to have shaped the complex distributions and genetic patterns observed in these willows. As such, evolutionary responses to Pleistocene climatic oscillations cannot be fully understood without integrating the ecological dimension.

## 5. Conclusions

Across all *Salix* species, our results reveal a consistent East–West genetic structure with a pre-Quaternary origin, likely driven by range shifts under changing environmental conditions. Eastern and western lineages also differ ecologically, although it remains unclear whether these differences drove or followed diversification. Demographic modelling supports repeated secondary contact during Quaternary interglacials, likely facilitated by the pioneer nature and rapid colonization capacity of willows. These recurrent reconnections likely prevented complete allopatric speciation, while ecological selection may have maintained lineage divergence despite gene flow. Together, these processes explain the persistence of distinct yet non-diverged *Salix* lineages within the Alps, with the most morphologically divergent recognized as sister species. More broadly, our study highlights that genetic structure in alpine species should not be interpreted solely through the lens of Pleistocene glacial cycles, as older evolutionary events and ecological differentiation can also contribute to shaping present-day biodiversity patterns. Integrating deep-time evolutionary history with ecological processes will therefore be essential for a more comprehensive understanding of diversification in mountain ecosystems.

## Figures and Tables

**Table 1:** List of *Salix* species included in the current study. Species already included in previous phylogeographic studies are indicated by a grey background.

Species	Ploidy Level	Allopolyploid Origins	Distribution	Elevation and Habitat	Substrate	Hybridization	Form
*S. alpina*	2×	-	Alps, Carpathians, Balkans	Subalpine screes, rocks, snowbeds (wet and light)	Basic	With *S. breviserrata*	Dwarf shrub
*S. appendiculata*	2×	-	Alps, Central European Mts., Dinarids	Montane to subalpine shrub communities (wet and partially shaded)	Indifferent	-	Shrub
*S. breviserrata*	2×	-	Cantabrian Mts., Alps	Subalpine to alpine screes, grasslands (wet and light)	Acidic to intermediate	With *S. alpina*	Dwarf shrub
*S. caesia*	4×	*S. purpurea* × *S. repens*	Alps	Subalpine wetlands	Mostly acidic	-	Shrub
*S. foetida*	2×	-	Alps	Subalpine to alpine wetlands	Mostly acidic	With *S. waldsteiniana*	Shrub
*S. helvetica*	2×	-	Alps, Tatra Mts.	Subalpine to alpine grasslands, snowbeds (wet and light)	Acidic (Siliceous)	-	Shrub
*S. laggeri*	4×	*S. appendiculata* × *S. caprea*	Alps	Montane to subalpine shrub communities (wet and light)	Acidic	-	Shrub
*S. retusa*	8×	Unknown	European mountain system	Alpine screes, snowbeds,	Intermediate to basic (calcareous)	-	Dwarf shrub
*S. serpillifolia*	2×	-	Alps	Alpine to nival grasslands, screes, rocks (sun-exposed)	Intermediate to basic (calcareous)	-	Dwarf shrub
*S. waldsteiniana*	2×	-	Alps, (Dinarids)	Subalpine to alpine shrub communities	Basic (calcareous)	With *S. foetida*	Shrub

**Table 2:** Results from the best performing demographic models and the corresponding unscaled parameter estimates. The test indicates whether origin or diversification models were tested. Source refers to the source population in the case of origin models, corresponding to population 1 in both models. Abbreviations: nuA = effective population size of the ancestral population; nu1 = effective population size of the source population before (up) and after (down) the expansion; nu2 = effective population size of the diverged population before (up) and after (down) the expansion; m = symmetric migration; m12/m21 = migration from population 1 to population 2 and population 2 to population 1, respectively; T1/T2/T3 = Time of period 1, 2, 3, respectively; s = founding fraction for the origin models. For more details, see [58,59]. Models and parameters correspond to those in Figure 3 and Figure 4.

Species	Test	Model	Source (1)	nuA	nu1	nu2	m12	m21	m	T1	T2	T3	s
*Salix alpina*- *Salix* *breviserrata*	Origin	founder_anc_sym_two_epoch	East	5.95	5.92	1.32			1.91	3.55	0.03		0.03
*Salix alpina*- *Salix* *breviserrata*	Diversification	sec_contact_sym_mig_size_three_epoch			8.24 28.6	3.27 25.9			5.46	2.28	0.64	0.15	
*Salix foetida*- *Salix* *waldsteiniana*	Origin	founder_anc_asym_two_epoch	East	0.41	0.35	1.76	5.44	0.21		0.71	0.01		0.15
*Salix foetida*- *Salix* *waldsteiniana*	Diversification	sec_contact_asym_mig_three_epoch			1.47	6.12	4.35	0.24		0.81	0.12	0.02	
*Salix* *appendiculata*	Origin	founder_sec_contact_asym_two_epoch	West	1.01	0.93	4.13	7.30	1.91		0.08	0.67		0.07
*Salix* *appendiculata*	Diversification	sec_contact_sym_mig_three_epoch			4.37	2.3			8.19	0.43	0.2	0.07	
*Salix* *serpillifolia*	Origin	founder_anc_asym_two_epoch	East	0.51	0.44	10.61	1.19	0.22		5.33	0.02		0.14
*Salix* *serpillifolia*	Diversification	sec_contact_sym_mig_size_three_epoch			0.71 4.11	26.3 3.71			0.2	0.6	0.71	0.18	
*Salix helvetica*	Origin	founder_anc_asym_two_epoch	West	3.29	3.19	3.46	5.72	2.23		5.01	0.15		0.03
*Salix helvetica*	Diversification	sec_contact_sym_mig_three_epoch			1.64	3.57			4.92	2.41	0.24	0.08	
*Salix laggeri*	Origin	founder_asym	West		1.37	19.68	12.08	1.87		0.92			0.07
*Salix laggeri*	Diversification	sec_contact_sym_mig_size			0.54 8.89	22.9 0.76			10.67	2.04	0.19		
*Salix retusa*	Origin	founder_sec_contact_asym_two_epoch	West	3.51	3.33	19.93	9.94	9.87		4.88	0.19		0.05
*Salix retusa*	Diversification	sec_contact_sym_mig_size			0.16 26	0.55 5.59			24.58	0.95	1.24		
*Salix caesia*	Origin	founder_sec_contact_asym_two_epoch	West	19.67	17.7	0.07	9.51	0.06		0.10	0.22		0.10
*Salix caesia*	Diversification	sec_contact_asym_mig_size			0.04 16.5	0.05 0.24	8.72	14.3		2.6	0.07		

**Table 3:** Ecological tests between intraspecific lineages. Corrected and uncorrected niche overlap, equivalency and similarity tests results indicated by *p*-values. Differences in niche optimum along both PC1 and PC2 are indicated by s = significant or ns = non-significant. Differences in niche breadth along both PC1 and PC2 are indicated by c = significant contraction of the eastern niche or ns = non-significant.

Species	*S. breviserrata* *S. alpina*	*S. foetida* *S. waldsteiniana*	*S. appendiculata*	*S. serpillifolia*	*S. helvetica*	*S. laggeri*	*S. retusa*	*S. caesia*
Range	West/East	West/East	West/East	West/East	West/East	West/East	West/East	West/East
Schoener’s D uncorrected	0.115	0.304	0.452	0.266	0.134	0.100	0.295	0.022
Schoener’s D corrected	0.138	0.306	0.541	0.229	0.118	0.181	0.382	0.077
Equivalency (Divergence)	0.010	1.000	1.000	0.564	0.158	0.663	1.000	0.653
Similarity 1-2 (Conservatism)	0.545	0.158	0.010	0.129	0.010	0.119	0.059	0.238
Similarity 2-1 (Conservatism)	0.455	0.149	0.010	0.040	0.020	0.119	0.069	0.238
Difference in niche optimum (PC1)	s	s	s	s	s	s	s	s
Difference in niche optimum (PC2)	s	s	s	s	s	s	s	s
Difference in niche breadth (PC1) W -> E	c	c	c	c	c	c	c	ns
Difference in niche breadth (PC2) W -> E	c	c	c	c	c	c	c	c

## Data Availability

Basic data supporting the findings are available within the manuscript and Supporting Information. All demultiplexed raw read data resulting from RAD sequencing were submitted to the National Centre for Biotechnology Information (NCBI) in the SequenceReadArchive (SRA) under different BioProject IDs (see Data S1). Sampling followed Nagoya regulations for the respective countries. Detailed locality for all accessions and summary of accessions included in the different analyses are provided in tabular format (Data S1).

## Supplementary Materials

The following supporting information can be downloaded at: https://www.mdpi.com/article/10.3390/biology15151299/s1, Data S1(Excel sheet): Location and sampling details of all accessions included in the article. Table S1: Summary of pipelines and parameters used to generate the different input files. Table S2: Total and projected number of individuals included for demographic modelling. Table S3: Name, description, and abbreviation of the environmental layers considered. Table S4: Number of occurrence records per species for ecological niche differentiation. Table S5: Summary of the best-performing replicate across models for the origin of the species pair *Salix alpina*-*Salix breviserrata*. Table S6: Summary of the best-performing replicate across models for the origin of the species pair *Salix foetida*-*Salix waldsteiniana*. Table S7: Summary of the best-performing replicate across models for the origin of the genetic groups of *Salix appendiculata.* Table S8: Summary of the best-performing replicate across models for the origin of the genetic groups of *Salix serpillifolia*. Table S9: Summary of the best-performing replicate across models for the origin of the genetic groups of *Salix helvetica*. Table S10: Summary of the best-performing replicate across models for the origin of the genetic groups of *Salix laggeri*. Table S11: Summary of the best-performing replicate across models for the origin of the genetic groups of *Salix retusa.* Table S12: Summary of the best-performing replicate across models for the origin of the genetic groups of *Salix caesia.* Table S13: Summary of the best-performing replicate across models for the diversification of the species pair *Salix alpina*-*Salix breviserrata*. Table S14: Summary of the best-performing replicate across models for the diversification of the species pair *Salix foetida*-*Salix waldsteiniana*. Table S15: Summary of the best-performing replicate across models for the diversification of the genetic groups of *Salix appendiculata.* Table S16: Summary of the best-performing replicate across models for the diversification of the genetic groups of *Salix serpillifolia*. Table S17: Summary of the best-performing replicate across models for the diversification of the genetic groups of *Salix helvetica*. Table S18: Summary of the best-performing replicate across models for the diversification of the genetic groups of *Salix laggeri*. Table S19: Summary of the best-performing replicate across models for the diversification of the genetic groups of *Salix retusa.* Table S20: Summary of the best-performing replicate across models for the diversification of the genetic groups of *Salix caesia.* Figure S1: Evanno analysis and genetic structure of the sister species when analyzed separately. Figure S2: Models used to test the origin of the genetic groups or species pairs. Figure S3: Models used to test the diversification mode of genetic groups or species pairs. Figure S4: Plot of the Pearson correlation coefficient between pairs of variables used for ecological niche differentiation. Figure S5: Evanno analysis showing the Delta K results. Figure S6: Results of the principal component analyses. Figure S7: Model fit evaluation for the test of the origin of the genetic groups or species pairs. Figure S8: Model fit evaluation for the test of the diversification of the genetic groups or species pairs. Figure S9: Summary of niche overlap between genetic groups or sister species pairs. Section S1: SNP filtering for different analyses. Section S2: Methodology description for goodness-of-fit tests. Section S3: Explanations for generation time estimates. Section S4: GBIF data sources. Section S5: Methodology description of occurrences data extraction and filtering. References [38,40,58,60,61,62,64,67,107,115,116,117,118,119,120,121] are cited in the supplementary materials.

## Abbreviation

The following abbreviation is used in this manuscript:

Mya	Million years ago
