# Supplementary material for: Miocene Evolutionary History Shaped the Phylogeography of Willow Species (Salix L.) in the European Alps
Source: Biology (Basel). 2026 Aug 5;15(15):1299. doi: 10.3390/biology15151299 (PMC13464956; doi:10.3390/biology15151299)
Supplement: Supplementary file 1 [file biology-15-01299-s001.zip › biology-4436231-supplementary.pdf]

Supplementary Materials

Miocene Evolutionary History Shaped the Phylogeography of Willow Species (*Salix* L.) in the European Alps

Loïc Pittet [1,2], Piotr Kosiński [3,4], Natascha D. Wagner [1] and Elvira Hörandl [1,*]

[1] Department of Systematics, Biodiversity, and Evolution of Plants (with Herbarium), University of Göttingen, 37073 Göttingen, Germany; loic.pittet@hotmail.com (L.P.); natascha.wagner@uni-goettingen.de (N.D.W.)
[2] Georg-August University School of Science (GAUSS), University of Göttingen, 37073 Göttingen, Germany
[3] Faculty of Agriculture, Horticulture and Biotechnology, Poznań University of Life Sciences, 60-637 Poznań, Poland; kosinski@man.poznan.pl
[4] Institute of Dendrology, Polish Academy of Sciences, 62-035 Kórnik, Poland
* Correspondence: elvira.hoerandl@biologie.uni-goettingen.de

The following Supplementary Information is available:

Data S1 (Excel sheet): Location and sampling details of all accessions included in the article.

Table S1: Summary of pipelines and parameters used to generate the different input files.

Table S2: Total and projected number of individuals included for demographic modelling.

Table S3: Name, description and abbreviation of the environmental layers considered.

Table S4: Number of occurrence records per species for ecological niche differentiation.

Table S5: Summary of the best-performing replicate across models for the origin of the species pair *Salix alpina* – *Salix breviserrata*.

Table S6: Summary of the best-performing replicate across models for the origin of the species pair *Salix foetida* – *Salix waldsteiniana*.

Table S7: Summary of the best-performing replicate across models for the origin of the genetic groups of *Salix appendiculata.*

Table S8: Summary of the best-performing replicate across models for the origin of the genetic groups of *Salix serpillifolia*.

Table S9: Summary of the best-performing replicate across models for the origin of the genetic groups of *Salix helvetica*.

Table S10: Summary of the best-performing replicate across models for the origin of the genetic groups of *Salix laggeri*.

Table S11: Summary of the best-performing replicate across models for the origin of the genetic groups of *Salix retusa.*

Table S12: Summary of the best-performing replicate across models for the origin of the genetic groups of *Salix caesia.*

Table S13: Summary of the best-performing replicate across models for the diversification of the species pair *Salix alpina* – *Salix breviserrata*.

Table S14: Summary of the best-performing replicate across models for the diversification of the species pair *Salix foetida* – *Salix waldsteiniana*.

Table S15: Summary of the best-performing replicate across models for the diversification of the genetic groups of *Salix appendiculata.*

Table S16: Summary of the best-performing replicate across models for the diversification of the genetic groups of *Salix serpillifolia*.

Table S17: Summary of the best-performing replicate across models for the diversification of the genetic groups of *Salix helvetica*.

Table S18: Summary of the best-performing replicate across models for the diversification of the genetic groups of *Salix laggeri*.

Table S19: Summary of the best-performing replicate across models for the diversification of the genetic groups of *Salix retusa.*

Table S20: Summary of the best-performing replicate across models for the diversification of the genetic groups of *Salix caesia.*

Figure S1: Evanno analysis and genetic structure of the sister species when analysed separately.

Figure S2: Models used to test the origin of the genetic groups or species pairs.

Figure S3: Models used to test the diversification mode of genetic groups or species pairs.

Figure S4: Plot of the Pearson correlation coefficient between pairs of variables used for ecological niche differentiation.

Figure S5: Evanno analysis showing the Delta K results.

Figure S6: Results of the principal component analyses.

Figure S7: Model fit evaluation for the test of the origin of the genetic groups or species pairs.

Figure S8: Model fit evaluation for the test of the diversification of the genetic groups or species pairs.

Figure S9: Summary of niche overlap between genetic groups or sister species pairs.

Section S1: SNP filtering for different analyses.

Section S2: Methodology description for goodness-of-fit tests.

Section S3: Explanations for generation time estimates.

Section S4: GBIF data sources.

Section S5: Methodology description of occurrences data extraction and filtering.

Table S1: Summary of pipelines and parameters used to generate the different input files for subsequent analyses. Abbreviations: N = number of individuals included in the specific dataset; M (denovo.pl) = number of mismatches allowed between stacks within individuals; m (denovo.pl) = Minimum number of reads to seed a stack; n (denovo.pl) = number of mismatches allowed between stacks between individuals; max-locus-stacks (denovo.pl) = maximum number of stacks at a single de novo locus; R (populations.pl) = minimum percentage of individuals across populations required to process a locus; max-obs-het (populations.pl) = maximum observed heterozygosity required to process a nucleotide site at a locus; write-single-snp (populations.pl) = restrict data analysis to only the first SNP per locus; hwe (plink) = exclude markers that failure the Hardy-Weinberg test at the specific value; geno (plink) = exclude SNPs with missing genotype rate superior than the specific value; mind (plink) = exclude individuals with missing genotype rate superior than the specific value; min_ind_with_reads (polyRAD) = minimum number of indiviuals with reads; min_ind_with_minor_allele (polyRAD) = minimum number of individuals with the minor allele; clust_threshold (ipyrad) = level of sequence similarity at which two sequences are identified as being homologous; max_alleles_consens (ipyrad) = maximum number of unique alleles allowed in consens reads after accounting for sequencing errors; min_samples_locus (ipyrad) = minimum number of samples that must have data at a given locus for it to be retained in the final data set. For more details, see [1-3].

Structure - PCA							
Species	pipeline	standard parameters (denovo_pl)	additional parameters (denovo_pl)	filters (populations_pl/polyRAD)	additional filters (PLINK)	N	SNPs
Diploid - single							
Salix alpina	denovo_pl + populations.pl + plink	"-M 4 -m 3 -n 4"		"-R 60 --max-obs-het 60 --write-single-snp"	"--hwe 0.05 --geno 0.1--mind 0.15"	40	48936
Salix appendiculata	denovo_pl + populations.pl + plink	"-M 4 -m 3 -n 4"		"-R 60 --max-obs-het 60 --write-single-snp"	"--hwe 0.05 --geno 0.1--mind 0.15"	44	37675
Salix breviserrata	denovo_pl + populations.pl + plink	"-M 4 -m 3 -n 4"		"-R 60 --max-obs-het 60 --write-single-snp"	"--hwe 0.05 --geno 0.1--mind 0.15"	30	48138
Salix foetida	denovo_pl + populations.pl + plink	"-M 4 -m 3 -n 4"		"-R 60 --max-obs-het 60 --write-single-snp"	"--hwe 0.05 --geno 0.1--mind 0.15"	43	45286
Salix helvetica	denovo_pl + populations.pl + plink	"-M 4 -m 3 -n 4"		"-R 60 --max-obs-het 60 --write-single-snp"	"--hwe 0.05 --geno 0.1--mind 0.15"	39	38448
Salix serpillifolia	denovo_pl + populations.pl + plink	"-M 4 -m 3 -n 4"		"-R 60 --max-obs-het 60 --write-single-snp"	"--hwe 0.05 --geno 0.1--mind 0.15"	52	28444

Salix waldsteiniana	denovo_pl + populations.pl + plink	"-M 4 -m 3 -n 4"		"-R 60 --max-obs-het 60 --write-single-snp"	"--hwe 0.05 --geno 0.1--mind 0.15"	42	44277
Diploid - hybridizing							
Salix alpina - Salix breviserrata	denovo_pl + populations.pl + plink	"-M 4 -m 3 -n 4"		"-R 60 --max-obs-het 60 --write-single-snp"	"--hwe 0.05 --geno 0.1--mind 0.15"	136	32796
Salix foetida – Salix waldsteiniana	denovo_pl + populations.pl + plink	"-M 4 -m 3 -n 4"		"-R 60 --max-obs-het 60 --write-single-snp"	"--hwe 0.05 --geno 0.1--mind 0.15"	131	26203
Polyploid - single							
Salix caesia	denovo_pl + polyRAD	"-M 4 -m 6 -n 4"	"--max_locus_stacks 4"	"min.ind.with.reads (60%) min.ind.with.minor.allele 4"		23	27205
Salix laggeri	denovo_pl + polyRAD	"-M 4 -m 6 -n 4"	"--max_locus_stacks 4"	"min.ind.with.reads (60%) min.ind.with.minor.allele 4"		21	22634
Salix retusa	denovo_pl + polyRAD	"-M 8 -m 6 -n 8"	"--max_locus_stacks 8"	"min.ind.with.reads (60%) min.ind.with.minor.allele 4"		57	30478

dadi

Species	pipeline	standard parameters (denovo_pl)	additional parameters (denovo_pl)	filters (populations_pl)	additional filters (PLINK; vcftools)	N	SNPs
Diploid - single							
Salix appendiculata	denovo_pl + populations.pl + plink	"-M 4 -m 3 -n 4"		"-R 60 --write-single-snp"	"--minDP 10 --maxDP 50 --minGQ 20"	28	60969
Salix helvetica	denovo_pl + populations.pl + plink	"-M 4 -m 3 -n 4"		"-R 60 --write-single-snp"	"--minDP 10 --maxDP 50 --minGQ 20"	28	61320
Salix serpillifolia	denovo_pl + populations.pl + plink	"-M 4 -m 3 -n 4"		"-R 60 --write-single-snp"	"--minDP 10 --maxDP 50 --minGQ 20"	29	64097

Diploid - hybridizing							
Salix alpina - Salix breviserrata	denovo_pl + populations.pl + plink	"-M 4 -m 3 -n 4"		"-R 60 --write-single-snp"	"--minDP 10 --maxDP 50 --minGQ 20"	72	59299
Salix foetida - Salix waldsteiniana	denovo_pl + populations.pl + plink	"-M 4 -m 3 -n 4"		"-R 60 --write-single-snp"	"--minDP 10 --maxDP 50 --minGQ 20"	67	56868
Polyploid - single							
Salix caesia	denovo_pl + populations.pl + plink	"-M 4 -m 6 -n 4"	"--max_locus_stacks 4"	"-R 60 --write-single-snp"	"--minDP 10 --maxDP 50 --minGQ 20"	18	62546
Salix laggeri	denovo_pl + populations.pl + plink	"-M 4 -m 6 -n 4"	"--max_locus_stacks 4"	"-R 60 --write-single-snp"	"--minDP 10 --maxDP 50 --minGQ 20"	21	64901
Salix retusa	denovo_pl + populations.pl + plink	"-M 8 -m 6 -n 8"	"--max_locus_stacks 8"	"-R 60 --write-single-snp"	"--minDP 10 --maxDP 50 --minGQ 20"	44	58241
RAxML - BEAST							
Species	pipeline	standard parameters (ipyrad)		additional parameters (ipyrad)		N	SNPs
All	ipyrad	"clust_threshold 0.85 max_alleles_consens 2 min_samples_locus 69"		"mindepth 6 max_shared_Hs_locus 0.5"		92	315876

Table S2: Number of individuals per group and projected sample sizes after downprojection with easySFS for downstream demographic inference in dadi.

Genetic group	Total number of ind.	Projected number of ind.
S. breviserrata	72	52
S. alpina	72	54
S. foetida	72	52
S. waldsteiniana	62	46
S. appendiculata (W)	34	26
S. appendiculata (E)	22	16
S. serpillifolia (W)	44	20
S. serpillifolia (E)	14	13
S. helvetica (W)	40	30
S. helvetica (E)	16	12
S. laggeri (W)	32	22
S. laggeri (E)	10	8
S. retusa (W)	56	36
S. retusa (E)	32	24
S. caesia (W)	28	22
S. caesia (E)	10	8

Table S3: Name, description and abbreviation of the environmental layers considered.

Name	Description	Abbreviation	Included
GLiM: global lithological map aggregated	Soil and topography	GLIM	Yes
SoilGrids: Cation exchange capacity	Soil and topography	CECSOL	Yes
SoilGrids: pH in KCL	Soil and topography	PHIKCL	Yes
SoilGrids: Silt content	Soil and topography	SLTPPT	Yes
SoilGrids: Clay content	Soil and topography	CLYPPT	Yes
SoilGrids: Coarse fragments	Soil and topography	CRFVOL	Yes
SoilGrids: Organic carbon density	Soil and topography	ORCDRC	Yes
SoilGrids: Available soil water capacity	Soil and topography	WWP	No
SoilGrids: Sand content	Soil and topography	SNDPPT	No
EU-DEM: Altitude	Soil and topography	ALTITUDE	No
EU-DEM: Slope	Soil and topography	SLOPE	No
CHELSA: Annual mean temperature	Bioclimate	bio1	No
CHELSA: Mean diurnal range	Bioclimate	bio2	No
CHELSA: Isothermality	Bioclimate	bio3	Yes
CHELSA: Temperature seasonality	Bioclimate	bio4	Yes
CHELSA: Max temperature of warmest month	Bioclimate	bio5	No
CHELSA: Min temperature of coldest month	Bioclimate	bio6	No
CHELSA: Temperature annual range	Bioclimate	bio7	No
CHELSA: Mean temperature of wettest quarter	Bioclimate	bio8	Yes
CHELSA: Mean temperature of driest quarter	Bioclimate	bio9	Yes
CHELSA: Mean temperature of warmest quarter	Bioclimate	bio10	No
CHELSA: Mean temperature of coldest quarter	Bioclimate	bio11	No
CHELSA: Annual precipitation sum	Bioclimate	bio12	No
CHELSA: Precipitation of wettest month	Bioclimate	bio13	No
CHELSA: Precipitation of warmest month	Bioclimate	bio14	No
CHELSA: Precipitation seasonality	Bioclimate	bio15	Yes
CHELSA: Precipitation of wettest quarter	Bioclimate	bio16	No
CHELSA: Precipitation of driest quarter	Bioclimate	bio17	No
CHELSA: Precipitation of warmest quarter	Bioclimate	bio18	No
CHELSA: Precipitation of coldest quarter	Bioclimate	bio19	No

Table S4: Number of occurrence records per species for ecological niche differentiation, classified to one of three geographic regions according to proximity to individuals with known genetic affiliation. The low number of records for *S.laggeri* and *S. caesia* in the eastern part are mostly due to wrong records in Tyrol and South Tyrol or older records that could not be confirmed.

Species	Area	Occurrences
S. breviserrata	West	273
S. breviserrata - S. alpina	Central	30
S. alpina	East	167
S. foetida	West	224
S. foetida - S. waldsteiniana	Central	142
S. waldsteiniana	East	240
S. appendiculata	West	393
S. appendiculata	Central	443
S. appendiculata	East	161
S. serpillifolia	West	171
S. serpillifolia	Central	92
S. serpillifolia	East	45
S. helvetica	West	640
S. helvetica	Central	20
S. helvetica	East	36
S. laggeri	West	114
S. laggeri	East	23
S. retusa	West	358
S. retusa	Central	662
S. retusa	East	198
S. caesia	West	357
S. caesia	Central	36
S. caesia	East	12
Total		4837

Table S5: Summary of the best-performing replicate across models for the origin of the species pair *Salix alpina* – *Salix breviserrata*. The comparison is based on Akaike Information Criterion. The parameters' estimates corresponding to the best replicate of the best model are given in Table **2**.

Model	log-likelihood	AIC	chi-squared	theta
founder_anc_sym_two_epoch	-2361.27	4732.54	496.15	2181.79
founder_asym	-2370.24	4754.48	363.13	6197.22
founder_anc_asym_two_epoch	-2375.14	4766.28	542.06	1358.85
founder_sym	-2379.56	4767.12	732.46	5694.38
founder_sec_contact_asym_two_epoch	-2383.27	4782.54	843.61	1763.66
vic_sec_contact_asym_mig	-2436.88	4883.76	815.32	10243.36
vic_sec_contact_sym_mig	-2456.22	4920.44	843.98	9630.24
vic_two_epoch_admix	-2500.56	5009.12	749.23	6484.98
founder_nomig_admix_two_epoch	-2509.61	5029.22	939.88	6650.23
vic_anc_sym_mig	-2527.36	5062.72	983.84	6494.84
vic_anc_asym_mig	-2527.91	5065.82	979.69	6507.3
founder_no_mig_two_epoch	-2551.05	5114.1	656.35	356.53
vic_no_mig_admix_late	-2627.15	5260.3	1657.41	6510.01
founder_nomig_admix_late	-2631.59	5271.18	1751.03	6511.2
vic_no_mig	-2638.61	5281.22	1289.01	6608.5
vic_no_mig_admix_early	-2638.62	5283.24	1294.19	6606.52
founder_nomig	-2638.83	5283.66	1265.13	6618.76
founder_nomig_admix_early	-2638.77	5285.54	1272.81	6615.58

Table S6: Summary of the best-performing replicate across models for the origin of the species pair *Salix foetida* – *Salix waldsteiniana*. The comparison is based on Akaike Information Criterion. The parameters' estimates corresponding to the best replicate of the best model are given in Table **2**.

Model	log-likelihood	AIC	chi-squared	theta
founder_anc_asym_two_epoch	-2155.81	4321.62	357.11	18002.34
founder_asym	-2159.64	4335.28	314.66	2503.04
founder_sec_contact_asym_two_epoch	-2214.69	4445.38	655.54	8382.51
founder_sym	-2318	4644	2216.52	3363.87
founder_anc_sym_two_epoch	-2319.19	4652.38	1684.8	4466.25
vic_sec_contact_asym_mig	-2544.17	5098.34	2551.43	20798.43
vic_sec_contact_sym_mig	-2571.39	5150.78	1974.44	16846.39
vic_two_epoch_admix	-2735.17	5478.34	2832.99	7167.44
founder_nomig_admix_two_epoch	-2735.59	5481.18	2701.53	6338.59
vic_anc_asym_mig	-2737.73	5485.46	2446.67	16276.78
vic_anc_sym_mig	-2894.94	5797.88	2549.4	9369.3
vic_no_mig_admix_late	-3246.66	6499.32	8657.28	5659.03
founder_nomig_admix_late	-3263.14	6534.28	8727.76	5673.15
vic_no_mig	-3391.26	6786.52	5251.61	5773.4
vic_no_mig_admix_early	-3391.26	6788.52	5254.19	5773.13
founder_nomig	-3391.84	6789.68	5346.97	5764.51
founder_nomig_admix_early	-3391.75	6791.5	5259.07	5773.92
founder_no_mig_two_epoch	-3391.54	6795.08	5270.26	1056.44

Table S7: Summary of the best-performing replicate across models for the origin of the genetic groups of *Salix appendiculata*. The comparison is based on Akaike Information Criterion. The parameters' estimates corresponding to the best replicate of the best model are given in Table **2**.

Model	log-likelihood	AIC	chi-squared	theta
founder_sec_contact_asym_two_epoch	-431.4	872.8	32.83	5776.77
founder_asym	-430.37	876.74	35.77	10810.61
founder_anc_asym_two_epoch	-434.15	884.3	40.97	4134.96
founder_anc_sym_two_epoch	-435.46	884.92	43.86	2897.02
founder_sym	-439.01	886.02	51.78	12925.7
founder_sym	-441.29	890.58	56.83	10845.13
founder_nomig_admix_two_epoch	-559.26	1128.52	312.7	6315.09
vic_two_epoch_admix	-589.07	1186.14	373.21	10543.21
vic_no_mig	-612.87	1229.74	425.84	6437.99
vic_no_mig_admix_early	-612.87	1231.74	425.87	6436.98
founder_nomig	-613.1	1232.2	426.62	6435.9
founder_nomig_admix_early	-612.88	1233.76	425.88	6437
founder_no_mig_two_epoch	-612.52	1237.04	424.63	328.48
vic_sec_contact_sym_mig	-627.66	1263.32	460.73	6520.99
vic_no_mig_admix_late	-634.18	1274.36	542.5	6861.85
founder_nomig_admix_late	-634.43	1276.86	541.3	6429.39
vic_anc_asym_mig	-653.16	1316.32	517.63	6591.87
vic_sec_contact_asym_mig	-760.31	1530.62	736.23	6522.49
vic_anc_sym_mig	-1002	2012	1201.69	6917.27

Table S8: Summary of the best-performing replicate across models for the origin of the genetic groups of *Salix serpillifolia*. The comparison is based on Akaike Information Criterion. The parameters' estimates corresponding to the best replicate of the best model are given in Table **2**.

Model	log-likelihood	AIC	chi-squared	theta
founder_anc_asym_two_epoch	-269.88	549.76	32.31	2851.78
founder_asym	-272.97	561.94	26.81	4660.1
founder_sec_contact_asym_two_epoch	-273.02	562.04	32.5	14775.93
vic_sec_contact_sym_mig	-281.66	571.32	51.52	25442.33
vic_sec_contact_asym_mig	-282.02	574.04	51.9	27636.1
founder_sym	-283.19	574.38	54.78	2243.61
founder_anc_sym_two_epoch	-292.23	598.46	73.42	1034.64
vic_anc_asym_mig	-300.62	611.24	93.87	23554.75
vic_anc_sym_mig	-320.39	648.78	137.59	4669.3
vic_two_epoch_admix	-343.23	694.46	182.96	5166.81
founder_nomig_admix_two_epoch	-348.75	707.5	196.92	5361.59
founder_nomig_admix_late	-405.87	819.74	324.93	4758.97
vic_no_mig_admix_late	-407.59	821.18	329.46	4814.5
vic_no_mig	-450.08	904.16	431.51	5203.9
vic_no_mig_admix_early	-450.08	906.16	431.71	5210.15
founder_nomig	-450.1	906.2	431.84	5212.08
founder_nomig_admix_early	-450.1	908.2	431.33	5196.08
founder_no_mig_two_epoch	-450.09	912.18	431.53	262.8

Table S9: Summary of the best-performing replicate across models for the origin of the genetic groups of *Salix helvetica*. The comparison is based on Akaike Information Criterion. The parameters' estimates corresponding to the best replicate of the best model are given in Table **2**.

Model	log-likelihood	AIC	chi-squared	theta
founder_anc_asym_two_epoch	-407.86	825.72	35.3	4651.54
founder_sym	-411.28	830.56	46	12110.23
founder_anc_sym_two_epoch	-408.77	831.54	36.58	2736.12
founder_asym	-408.67	833.34	34.49	6620.31
founder_sym	-415.27	838.54	54.92	10153.09
founder_sec_contact_asym_two_epoch	-411.71	839.42	44.31	949.44
vic_two_epoch_admix	-533.33	1074.66	303.59	15224.15
vic_no_mig_admix_late	-540.2	1086.4	370.01	7633.25
founder_nomig_admix_late	-540.84	1089.68	373.12	7633.71
founder_nomig_admix_two_epoch	-552.55	1115.1	346.72	7825.02
vic_sec_contact_sym_mig	-573.04	1154.08	381.48	7789.36
vic_sec_contact_asym_mig	-582.03	1174.06	400.4	8735.34
founder_no_mig_two_epoch	-605.34	1222.68	439.79	399.09
vic_no_mig	-614.58	1233.16	475.69	7891.81
vic_anc_asym_mig	-612.11	1234.22	472.03	7893.36
vic_no_mig_admix_early	-614.59	1235.18	475.94	7889.71
founder_nomig	-614.61	1235.22	475.87	7891.43
founder_nomig_admix_early	-614.59	1237.18	475.89	7890.41
vic_anc_sym_mig	-641.05	1290.1	520.17	8033.19

Table S10: Summary of the best-performing replicate across models for the origin of the genetic groups of *Salix laggeri*. The comparison is based on Akaike Information Criterion. The parameters' estimates corresponding to the best replicate of the best model are given in Table **2**.

Model	log-likelihood	AIC	chi-squared	theta
founder_asym	-264.45	538.9	178.48	11827.4
founder_sym	-275.86	559.72	206.94	9648.29
founder_sec_contact_asym_two_epoch	-273.58	563.16	203.05	6232.81
founder_anc_asym_two_epoch	-279.74	575.48	211.68	2905.37
founder_anc_sym_two_epoch	-288.21	590.42	226.64	3517.97
vic_no_mig_admix_late	-406.51	819.02	452.18	7247.91
founder_nomig_admix_late	-406.44	820.88	452.05	6409.04
founder_nomig_admix_two_epoch	-405.9	821.8	450.01	6430.66
vic_no_mig	-480.68	965.36	669.31	6414.53
vic_two_epoch_admix	-479.61	967.22	658.88	6444.37
vic_no_mig_admix_early	-480.68	967.36	669.22	6414.87
founder_nomig	-480.69	967.38	669.06	6415.53
founder_nomig_admix_early	-480.69	969.38	668.93	6416.06
founder_no_mig_two_epoch	-480.33	972.66	668.68	453.48
vic_anc_asym_mig	-517.89	1045.78	715.39	6591.28
vic_anc_sym_mig	-526.32	1060.64	729.81	6613.3
vic_sec_contact_sym_mig	-574.94	1157.88	801.75	6657.46
vic_sec_contact_asym_mig	-576.01	1162.02	804.42	6678.04

Table S11: Summary of the best-performing replicate across models for the origin of the genetic groups *of Salix retusa*. The comparison is based on Akaike Information Criterion. The parameters' estimates corresponding to the best replicate of the best model are given in Table **2**.

Model	log-likelihood	AIC	chi-squared	theta
founder_sec_contact_asym_two_epoch	-1184.61	2385.22	1623.8	387.6
founder_anc_sym_two_epoch	-1509.8	3033.6	3181.01	527.12
founder_anc_asym_two_epoch	-1573.69	3163.38	3417.62	207.07
founder_sym	-1583.54	3175.08	3519.91	2524.23
founder_asym	-1591.2	3192.4	3608.84	2527.28
vic_no_mig_admix_late	-1610.71	3227.42	3201.01	3334.44
founder_nomig_admix_late	-1610.99	3229.98	3200.52	2157.24
founder_no_mig_two_epoch	-1650.15	3312.3	3307.67	108.64
founder_nomig_admix_two_epoch	-1746.64	3503.28	3562.14	2045.24
founder_nomig	-1856.95	3719.9	3850.04	2035.16
founder_nomig_admix_early	-1857.11	3722.22	3850.47	2035.19
vic_no_mig	-2113.68	4231.36	4511.53	2091.5
vic_no_mig_admix_early	-2113.52	4233.04	4511.07	2091.48
vic_two_epoch_admix	-2115.88	4239.76	4517.57	4109.76
vic_sec_contact_sym_mig	-2499.64	5007.28	5529.65	2178.41
vic_anc_sym_mig	-2543.12	5094.24	5649.97	2190.44
vic_sec_contact_asym_mig	-2794.79	5599.58	6306.49	2168.65
vic_anc_asym_mig	-3229.71	6469.42	7568.35	2321.31

Table S12: Summary of the best-performing replicate across models for the origin of the genetic groups of *Salix caesia*. The comparison is based on Akaike Information Criterion. The parameters' estimates corresponding to the best replicate of the best model are given in Table **2**.

Model	log-likelihood	AIC	chi-squared	theta
founder_sec_contact_asym_two_epoch	-6855.82	13727.64	15830.05	7198.88
vic_sec_contact_sym_mig	-7166.17	14340.34	16816.75	1275.59
vic_sec_contact_asym_mig	-7438.45	14886.9	17797.53	1356.76
founder_asym	-7438.81	14887.62	18015.48	32739.95
founder_anc_sym_two_epoch	-7953.25	15920.5	19625.46	734.33
founder_anc_asym_two_epoch	-8033.09	16082.18	20051.28	1428.99
vic_anc_asym_mig	-8048.19	16106.38	19817.56	20434.54
founder_nomig_admix_two_epoch	-8174.85	16359.7	20442.1	10579.66
vic_two_epoch_admix	-8371.32	16750.64	21174.17	10260.75
founder_sym	-8385.97	16779.94	21940.95	9164.04
founder_nomig_admix_late	-8447.71	16903.42	21633.45	8792.08
vic_no_mig_admix_late	-8452.32	16910.64	21617.53	8821.2
vic_no_mig	-8534.34	17072.68	21882.93	9008.31
vic_no_mig_admix_early	-8534.34	17074.68	21882.99	9007.25
founder_nomig	-8534.51	17075.02	21883.03	9011.72
vic_anc_sym_mig	-8534.38	17076.76	21883.06	9007.94
founder_nomig_admix_early	-8534.39	17076.78	21882.76	9012.39
founder_no_mig_two_epoch	-8534.41	17080.82	21882.7	520.82

Table S13: Summary of the best-performing replicate across models for the diversification of the species pair *Salix alpina* – *Salix breviserrata*. The comparison is based on Akaike Information Criterion. The parameters' estimates corresponding to the best replicate of the best model are given in Table **2**.

Model	log-likelihood	AIC	chi-squared	theta
sec_contact_sym_mig_size_three_epoch	-2346.26	4708.52	276.92	6024.18
anc_sym_mig	-2349.37	4708.74	318.98	5995.71
anc_asym_mig_size	-2346.63	4709.26	356.47	6008.11
sec_contact_asym_mig_three_epoch	-2347.64	4709.28	321.92	6033.61
anc_asym_mig	-2350.96	4713.92	284.31	5988.49
sec_contact_asym_mig	-2356.14	4724.28	444.96	5559.14
anc_sym_mig_size	-2356.52	4727.04	323.52	6061.47
sec_contact_asym_mig_size_three_epoch	-2356.83	4731.66	377.13	799.04
sec_contact_asym_mig_size	-2359.58	4735.16	411.74	1272.19
asym_mig_size	-2361.04	4738.08	494.43	3649.07
asym_mig	-2368.44	4746.88	623.31	4339.33
asym_mig_twoepoch	-2368.23	4752.46	517.16	3468.9
sym_mig_twoepoch	-2370.35	4752.7	692.7	3843.97
sym_mig_size	-2370.33	4754.66	689.88	4088.19
sec_contact_sym_mig_size	-2370.64	4755.28	640.64	4025.33
sec_contact_sym_mig_three_epoch	-2376.68	4765.36	517.49	1407.83
sym_mig	-2388.79	4785.58	864.49	1813.97
no_mig	-2638.35	5282.7	1286.97	6608
no_mig_size	-2638.62	5289.24	1287.57	6609.16

Table S14: Summary of the best-performing replicate across models for the diversification of the species pair *Salix foetida* – *Salix waldsteiniana*. The comparison is based on Akaike Information Criterion. The parameters' estimates corresponding to the best replicate of the best model are given in Table **2**.

Model	log-likelihood	AIC	chi-squared	theta
sec_contact_asym_mig_three_epoch	-2131.15	4276.3	297.77	4442.23
anc_asym_mig	-2134.77	4281.54	282.78	4378.61
sec_contact_asym_mig_size	-2139.89	4295.78	317.97	4311.77
asym_mig	-2144.5	4299	284.32	4305.31
asym_mig_size	-2155.49	4326.98	415.37	3989.26
sec_contact_sym_mig_size_three_epoch	-2167.42	4350.84	360.37	3547.47
anc_asym_mig_size	-2169.89	4355.78	396.46	4424.33
anc_sym_mig_size	-2177.39	4368.78	409.63	4307.86
sec_contact_sym_mig_three_epoch	-2178.55	4369.1	442.84	4443.76
sec_contact_asym_mig	-2183.51	4379.02	365.67	4109.04
asym_mig_twoepoch	-2186.23	4388.46	395.75	3777.31
sym_mig_twoepoch	-2193.89	4399.78	503.49	4351.33
anc_sym_mig	-2195.27	4400.54	536.31	4296.01
sec_contact_asym_mig_size_three_epoch	-2214.87	4447.74	834.57	3350.63
sym_mig_size	-2247.21	4508.42	1307.66	5936.26
sec_contact_sym_mig_size	-2248.65	4511.3	1002.89	4247.44
sec_contact_sym_mig	-2258.17	4526.34	973.34	4121.09
sym_mig	-2262.05	4532.1	1328.68	3368.06
no_mig	-3391.27	6788.54	5258.29	5772.69
no_mig_size	-3392.06	6796.12	5280.24	5772.42

Table S15: Summary of the best-performing replicate across models for the diversification of the genetic groups of *Salix appendiculata*. The comparison is based on Akaike Information Criterion. The parameters' estimates corresponding to the best replicate of the best model are given in Table **2**.

Model	log-likelihood	AIC	chi-squared	theta
sec_contact_sym_mig_three_epoch	-429.22	870.44	29.73	5650.13
sec_contact_asym_mig_size_three_epoch	-430.34	868.68	29.8	5642.81
sec_contact_sym_mig	-430.44	870.88	32.92	5640.44
anc_asym_mig	-429.79	871.58	31.4	5652.91
asym_mig	-430.79	871.58	33.93	5620.98
anc_sym_mig	-430.8	871.6	32.54	5603.71
sym_mig	-429.29	872.58	32.89	5609.57
sym_mig_twoepoch	-430.46	872.92	33.29	5593.79
anc_asym_mig_size	-428.81	873.62	29.11	5630.94
sec_contact_sym_mig_size_three_epoch	-429.09	874.18	29.31	2191.83
sym_mig_size	-430.74	875.48	33.11	5614.83
sec_contact_asym_mig_size	-429.79	875.58	31.01	5606.97
sec_contact_asym_mig_three_epoch	-429.54	877.08	30.9	1475.53
anc_sym_mig_size	-431.74	877.48	35.49	5592.3
asym_mig_size	-430.95	877.9	33.75	5180.96
asym_mig_twoepoch	-432.62	881.24	37.08	12586.44
sec_contact_sym_mig_size	-435.23	884.46	43.23	2846.98
sec_contact_asym_mig	-455.93	923.86	85.96	2032.86
no_mig	-612.87	1231.74	425.83	6438.69
no_mig_size	-612.91	1237.82	425.99	6436.43

Table **S16**: Summary of the best-performing replicate across models for the diversification of the genetic groups of *Salix serpillifolia*. The comparison is based on Akaike Information Criterion. The parameters' estimates corresponding to the best replicate of the best model are given in Table **2**.

Model	log-likelihood	AIC	chi-squared	theta
sec_contact_sym_mig_size_three_epoch	-265.95	547.9	18.95	4623.04
sym_mig	-270.52	549.04	27.94	4070.74
sym_mig_size	-268.2	550.4	23.59	4625.19
sec_contact_sym_mig	-270.61	551.22	28.12	4025.38
asym_mig	-271.01	552.02	29.17	4310.4
anc_asym_mig	-270.13	552.26	28.1	4150.96
anc_sym_mig	-271.22	552.44	29.42	3188.49
asym_mig_twoepoch	-268.53	553.06	24.15	4462.1
sec_contact_sym_mig_size	-270.44	554.88	27.77	4084.59
sec_contact_sym_mig_three_epoch	-271.51	555.02	30.26	4045.02
sym_mig_twoepoch	-272.31	556.62	31.96	4045.26
sec_contact_asym_mig_three_epoch	-271.32	556.64	30.42	4151.66
sec_contact_asym_mig_size	-271.4	558.8	30.31	4206.14
asym_mig_size	-271.65	559.3	29.98	3880.11
sec_contact_asym_mig_size_three_epoch	-270.8	559.6	29.01	4471.94
anc_asym_mig_size	-272.03	560.06	30.79	2098.44
sec_contact_asym_mig	-278.22	568.44	44.22	3050.37
anc_sym_mig_size	-283.21	580.42	54.54	2436.37
no_mig	-448.21	902.42	425.23	5017.44
no_mig_size	-448.12	908.24	424.92	5006.64

Table S17: Summary of the best-performing replicate across models for the diversification of the genetic groups of *Salix helvetica*. The comparison is based on Akaike Information Criterion. The parameters' estimates corresponding to the best replicate of the best model are given in Table **2**.

Model	log-likelihood	AIC	chi-squared	theta
sec_contact_sym_mig_three_epoch	-407.9	827.28	32.9	3319.97
asym_mig	-408.73	827.46	36.62	6077.28
anc_sym_mig	-408.91	827.82	34.69	6050.42
sec_contact_asym_mig	-408.03	828.06	36.05	5964.14
anc_asym_mig	-408.39	828.78	33.77	6145.09
sec_contact_asym_mig_three_epoch	-408.2	830.4	34.3	6365.88
anc_asym_mig_size	-408.21	832.42	33.32	5981.61
sec_contact_sym_mig	-411.36	832.72	47.93	4646.83
anc_sym_mig_size	-409.69	833.38	37.03	6150.41
asym_mig_twoepoch	-408.96	833.92	37.82	6415.31
sec_contact_asym_mig_size_three_epoch	-408.58	835.16	34.46	8944.97
sym_mig	-414.21	836.42	52.96	5351.17
asym_mig_size	-410.53	837.06	43.32	6114.74
sym_mig_size	-413.6	841.2	51.58	5608.05
sym_mig_twoepoch	-414.86	841.72	52.96	3358.25
sec_contact_sym_mig_size	-413.97	841.94	54.27	999.25
sec_contact_asym_mig_size	-413.78	843.56	53.01	5602
no_mig	-614.56	1235.12	475.43	7893.34
no_mig_size	-614.67	1241.34	476.15	7891.31

Table S18: Summary of the best-performing replicate across models for the diversification of the genetic groups of *Salix laggeri*. The comparison is based on Akaike Information Criterion. The parameters' estimates corresponding to the best replicate of the best model are given in Table **2**.

Model	log-likelihood	AIC	chi-squared	theta
sec_contact_sym_mig_size	-243.02	500.04	131.36	3432.94
sec_contact_asym_mig_size	-243.99	503.98	132.98	5954.46
sym_mig_twoepoch	-246.76	505.52	141.69	5459.57
sym_mig	-248.86	505.72	146.43	5504
sec_contact_asym_mig	-247.25	506.5	141.92	5523.16
asym_mig	-248.71	507.42	145.21	5492.13
sec_contact_sym_mig	-249.57	509.14	147.74	5436.8
asym_mig_size	-247.93	511.86	146.75	28557.59
anc_asym_mig	-251.21	514.42	149.55	5484.56
anc_sym_mig	-252.32	514.64	149.38	5570.13
sec_contact_asym_mig_size_three_epoch	-250.94	519.88	146.88	6562.86
anc_sym_mig_size	-254.47	522.94	155.97	5570
asym_mig_twoepoch	-255.53	527.06	159	5563.4
sec_contact_sym_mig_size_three_epoch	-256.99	529.98	155.37	1089.25
sym_mig_size	-259.3	532.6	165.62	2693.89
sec_contact_asym_mig_three_epoch	-265.41	544.82	178.29	1094.58
anc_asym_mig_size	-276.83	569.66	215.63	5216.14
no_mig	-480.68	967.36	668.82	6416.48
no_mig_size	-480.68	973.36	668.96	6415.92

Table S19: Summary of the best-performing replicate across models for the diversification of the genetic groups of *Salix retusa*. The comparison is based on Akaike Information Criterion. The parameters' estimates corresponding to the best replicate of the best model are given in Table **2**.

Model	log-likelihood	AIC	chi-squared	theta
sec_contact_sym_mig_size	-780.28	1574.56	692.52	1306.64
sec_contact_sym_mig_size_three_epoch	-816.9	1649.8	820.33	631.43
sec_contact_sym_mig	-965.41	1940.82	1264.09	161.31
anc_asym_mig	-973.57	1959.14	1300.25	316.63
sec_contact_asym_mig	-1056.03	2124.06	1308.3	123.22
anc_sym_mig	-1129.29	2268.58	2450.35	1050.29
sym_mig_twoepoch	-1233.25	2478.5	2781.11	1374.51
asym_mig_size	-1245.51	2507.02	2497.47	2895.31
sec_contact_asym_mig_size	-1269.16	2554.32	2544.04	3429.66
sym_mig_size	-1298.23	2610.46	2792.87	1901.13
sec_contact_asym_mig_size_three_epoch	-1328.46	2674.92	2925.8	1884.47
asym_mig_twoepoch	-1339.6	2695.2	3000.74	1644.4
asym_mig	-1346.83	2703.66	3003.84	1663.14
sec_contact_sym_mig_three_epoch	-1352.13	2716.26	3052.23	1644.39
anc_sym_mig_size	-1374.32	2762.64	3148.84	1629.22
anc_asym_mig_size	-1393.25	2802.5	3249.08	1622.07
sec_contact_asym_mig_three_epoch	-1412.86	2839.72	3155.31	1698.27
no_mig	-1623.25	3252.5	3235.94	1981.59
no_mig_size	-1692.52	3397.04	3421.41	1997.38

Table S20: Summary of the best-performing replicate across models for the diversification of the genetic groups of *Salix caesia*. The comparison is based on Akaike Information Criterion. The parameters' estimates corresponding to the best replicate of the best model are given in Table **2**.

Model	log-likelihood	AIC	chi-squared	theta
sec_contact_asym_mig_size	-6286.3	12588.6	15730.02	3250.73
sec_contact_asym_mig_size_three_epoch	-6314.64	12647.28	14343.21	458.52
sec_contact_asym_mig	-6487.07	12986.14	15492.18	415.25
sec_contact_sym_mig_size_three_epoch	-6498.79	13013.58	14212.75	10131.87
sec_contact_sym_mig_size	-6604.61	13223.22	15835.96	534.21
sec_contact_sym_mig	-6777.55	13565.1	16540.71	410.98
asym_mig	-7088.56	14187.12	16447.84	56989.35
sym_mig_twoepoch	-7341.36	14694.72	17221.31	1844.13
asym_mig_twoepoch	-7371.61	14759.22	17482.74	35753.1
sec_contact_sym_mig_three_epoch	-7450.42	14912.84	17718.85	464.29
sym_mig_size	-8105.89	16225.78	20291.43	1591.72
asym_mig_size	-8489.07	16994.14	21722.34	8852.05
sec_contact_asym_mig_three_epoch	-8517.63	17049.26	21813.07	9552.39
no_mig_size	-8518.87	17049.74	21894.34	8957.15
anc_asym_mig_size	-8517.15	17050.3	21792.32	16227.68
no_mig	-8528.91	17063.82	21886.94	9013.86
anc_sym_mig	-8529.02	17068.04	21886.51	8999.33
anc_asym_mig	-8528.94	17069.88	21886.59	9001.26
sym_mig	-8531.72	17071.44	21885.27	8978.95
anc_sym_mig_size	-8532.69	17079.38	21883.1	9010.16

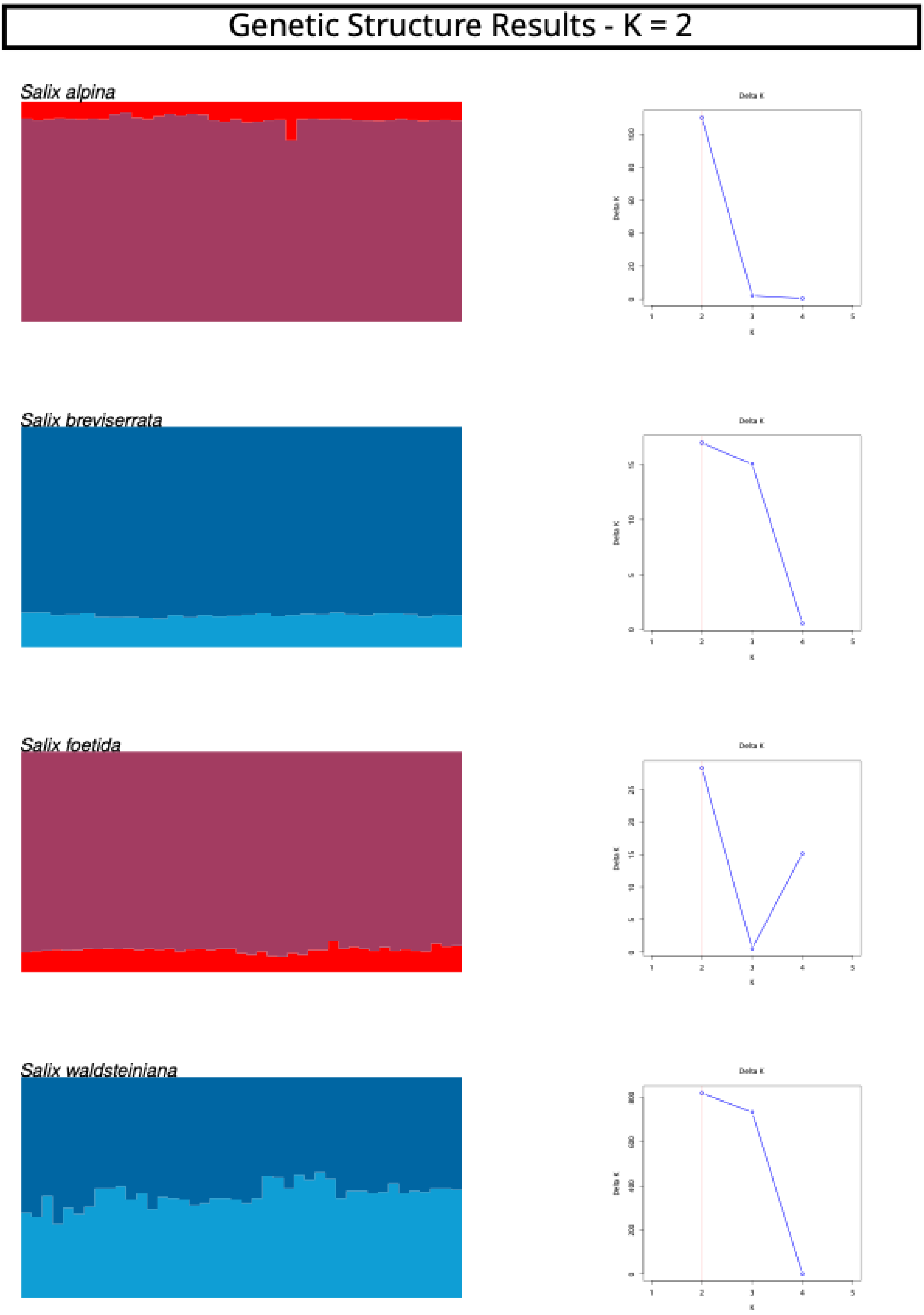

Figure S1: Evanno analysis and genetic structure of the sister species when analysed separately. Results from the Delta K analysis (left). Genetic structure of each species independently for K=2.

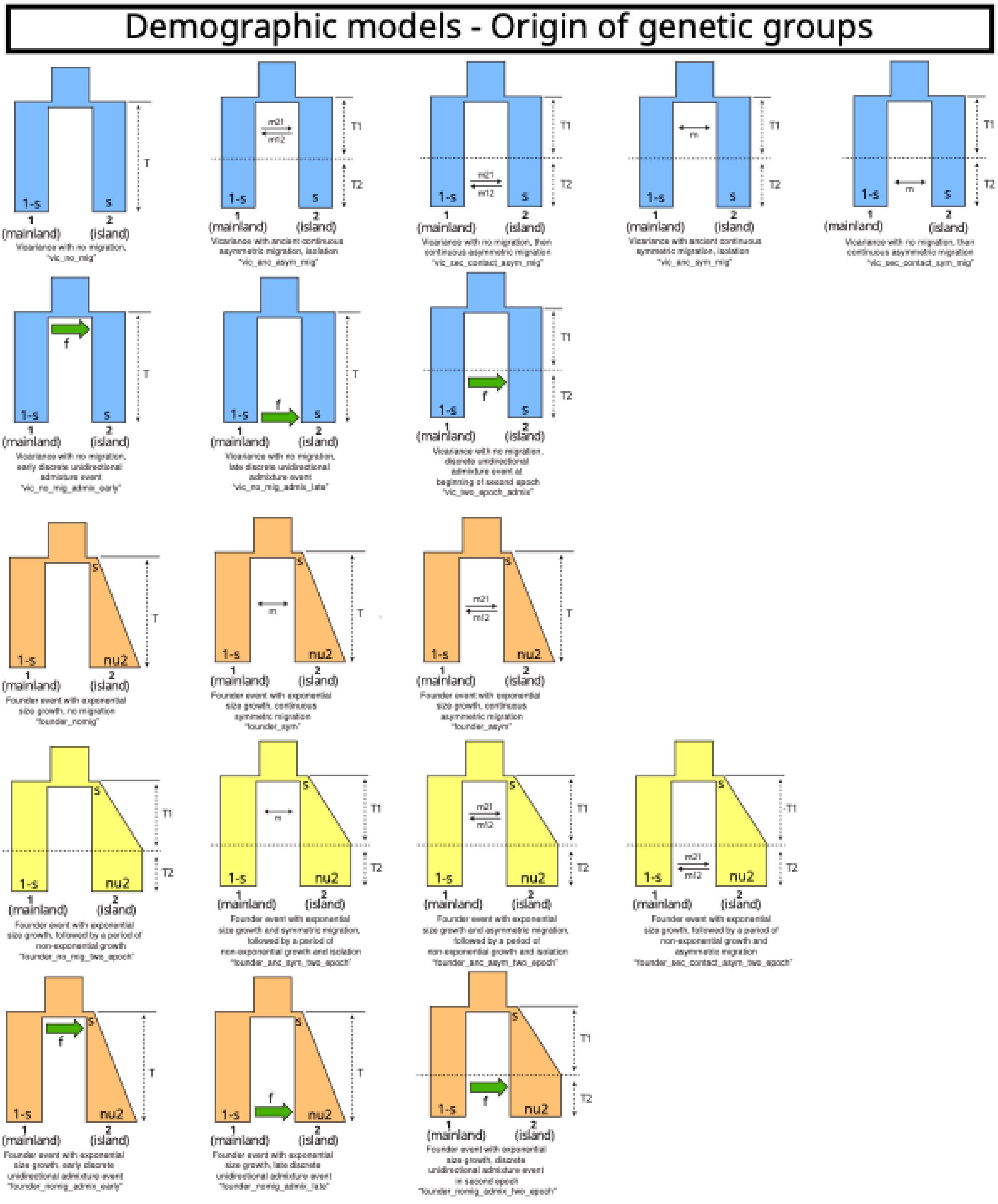

Figure S2: Models used to test the origin of the genetic groups or species pairs. Models in blue and orange describe variants of vicariance and founder events, respectively, and were taken from [4]. Models in yellow also represent founder events but were defined in [5].

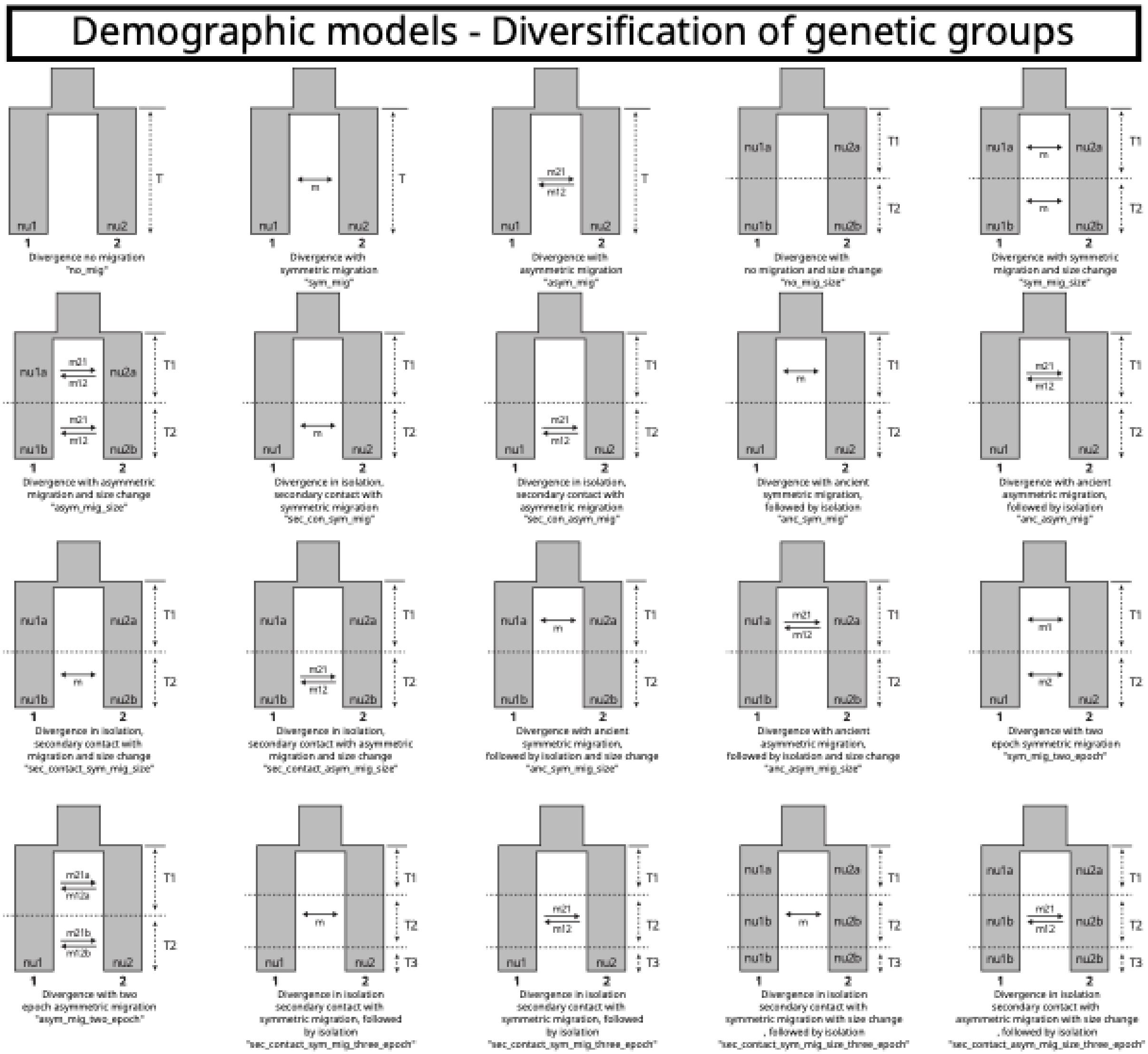

Figure S3: Models used to test the diversification mode of genetic groups or species pairs. Models were taken from [4].

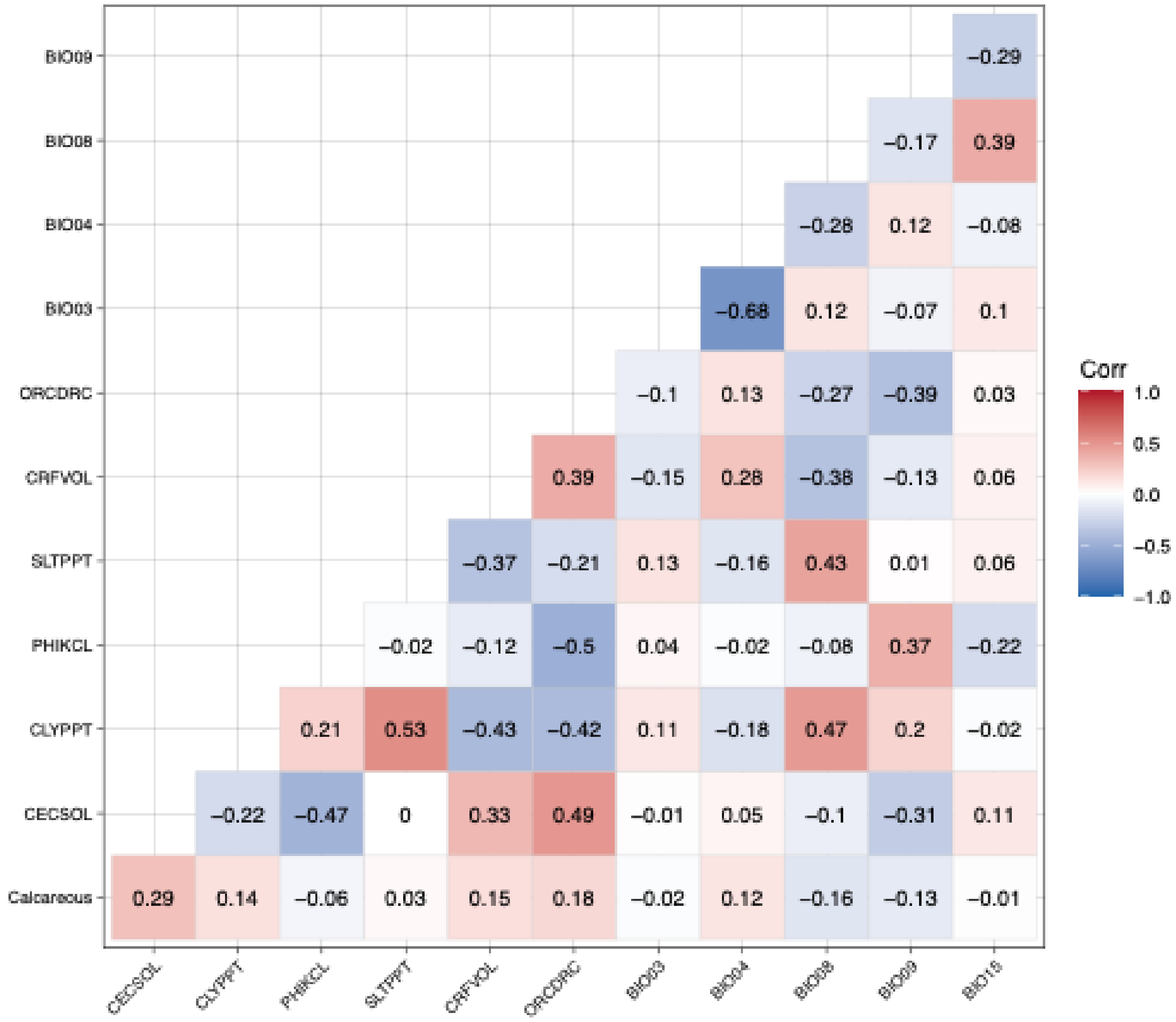

Figure S4: Plot of the Pearson correlation coefficient between pairs of variables used for ecological niche differentiation. Abbreviations: CECSOL = Cation exchange capacity, CLYPPT = Clay content , PHIKCL = pH in KCL, SLTPPT = Silt content, CRFVOL = Coarse fragments, ORCDRC = Organic carbon density, BIO03 = Isothermality, BIO04 = Temperature seasonality, BIO08 = Mean temperature of wettest quarter, BIO09 = Mean temperature of driest quarter, BIO15 = Precipitation seasonality. Variable sources are defined in Supporting Information Table **S2**.

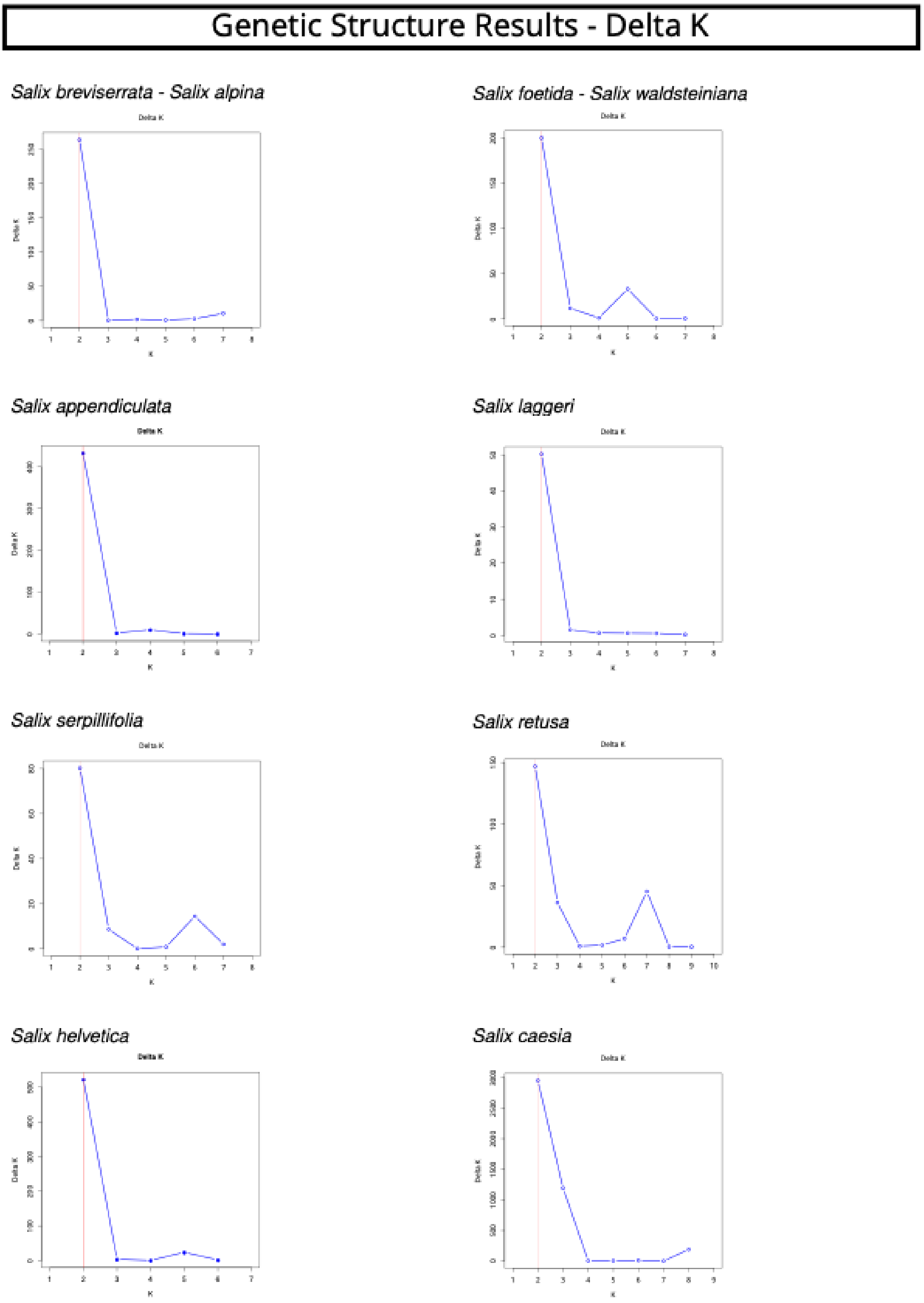

Figure S5: Evanno analysis showing the Delta K results for the two sister species pairs *Salix alpina, Salix breviserrata and Salix foetida*, *Salix waldsteiniana*, for the three other diploid species *Salix appendiculata*, *Salix serpillifolia* and *Salix helvetica*, and for the three polyploids *Salix laggeri*, *Salix retusa* and *Salix caesia*.

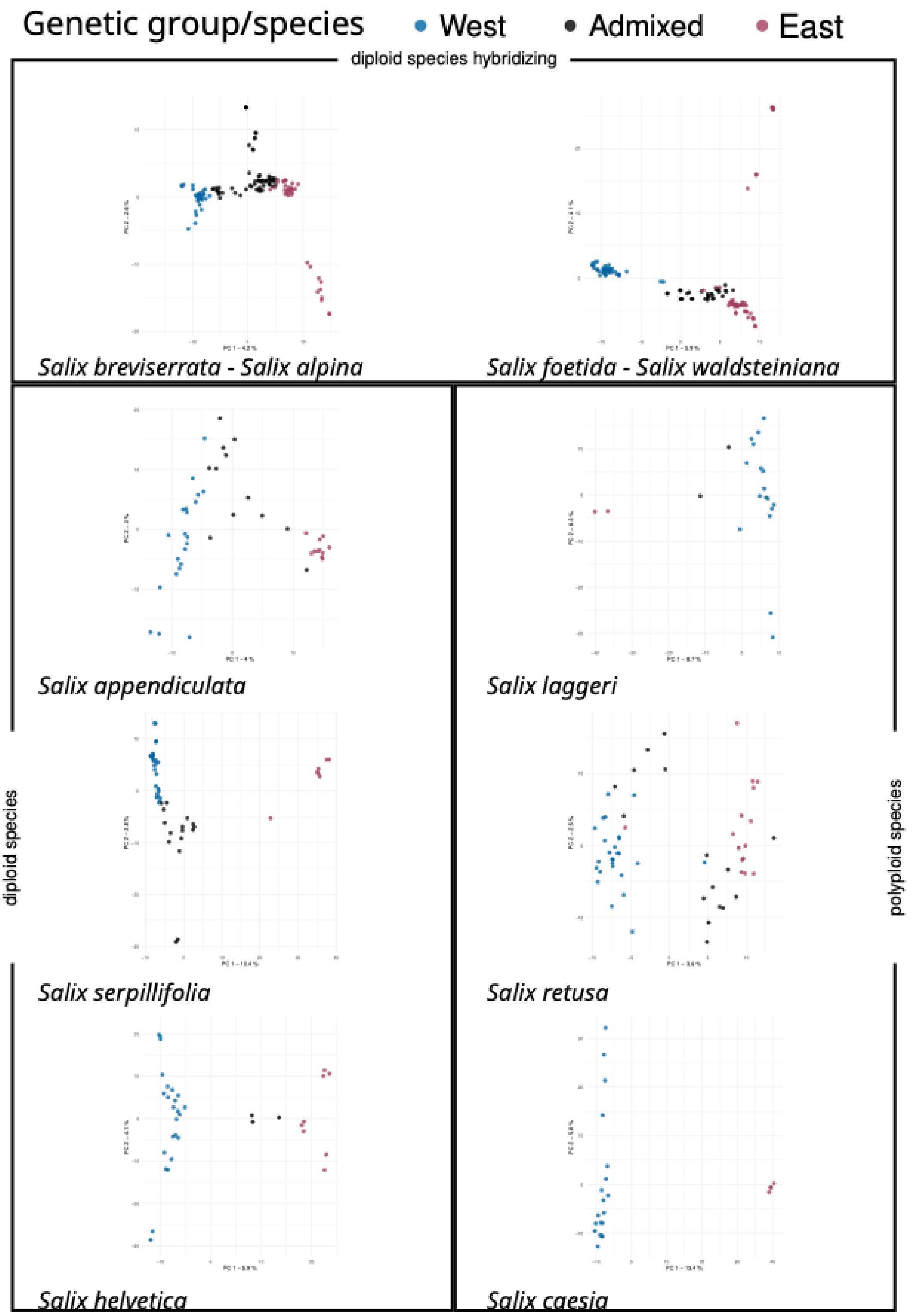

Figure S6: Principal component analyses of the two sister species pairs *Salix alpina*, *Salix breviserrata* and *Salix foetida*, *Salix waldsteiniana*, for the three other diploid species *Salix appendiculata*, *Salix serpillifolia* and *Salix helvetica*, and for the three polyploids *Salix laggeri*, *Salix retusa* and *Salix caesia*. Colours were defined based on STRUCTURE results at K=2, assigning individuals with <10% admixture to their respective genetic groups (blue for the western group and red for the eastern group) while individuals with >10% admixture were classified as admixed individuals (black).

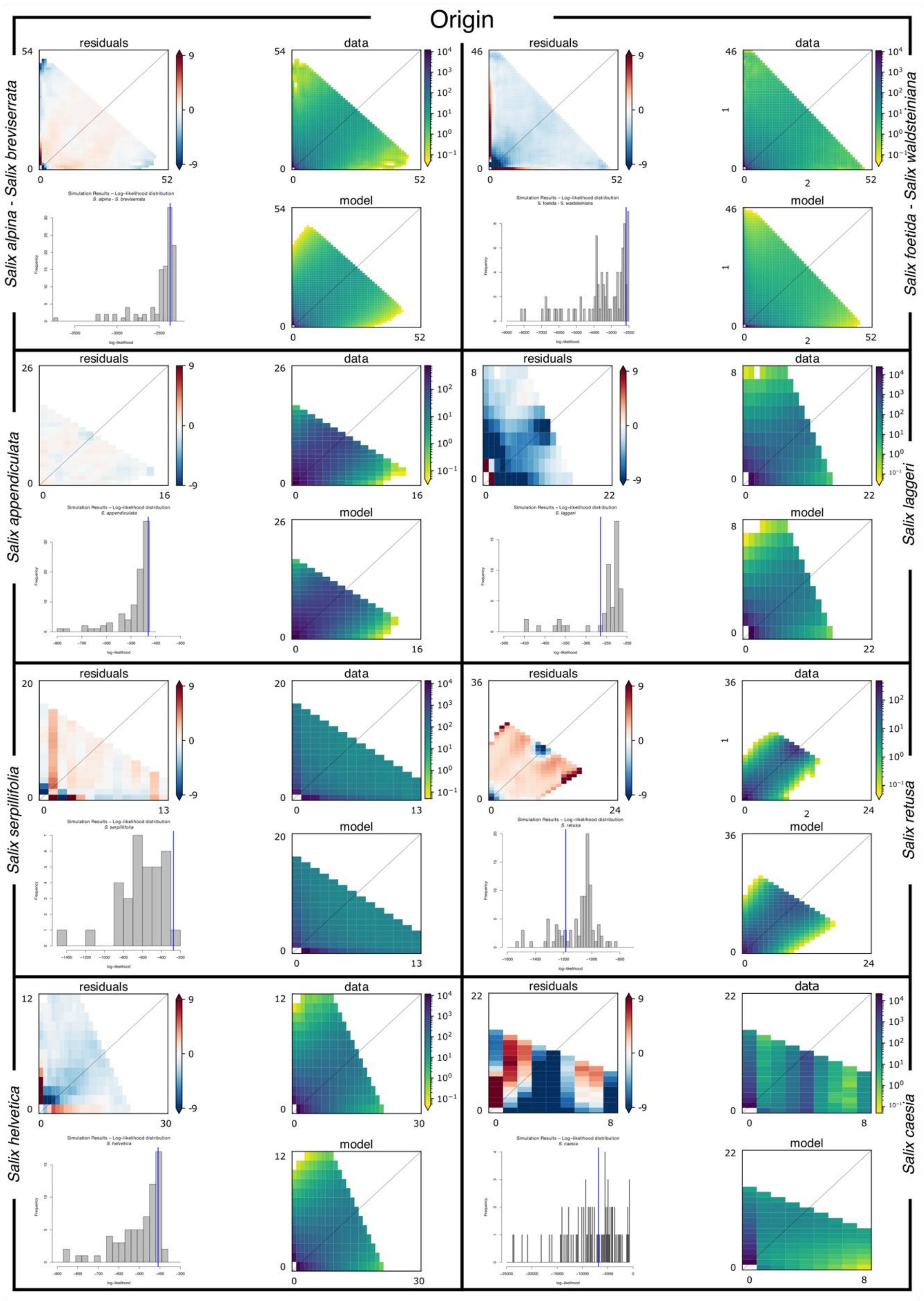

Figure S7: Evaluation of the best models for the origin of the genetic groups (Figure **3**). For the best model of each species, the empirical joint site frequency spectrum (top right), the joint site frequency spectrum inferred by the model (bottom right), the corresponding residuals (top left) between empirical and modeled data, and the estimated log-likelihood (blue line) and their distribution from the goodness-of-fit test (100 replicates) are displayed.

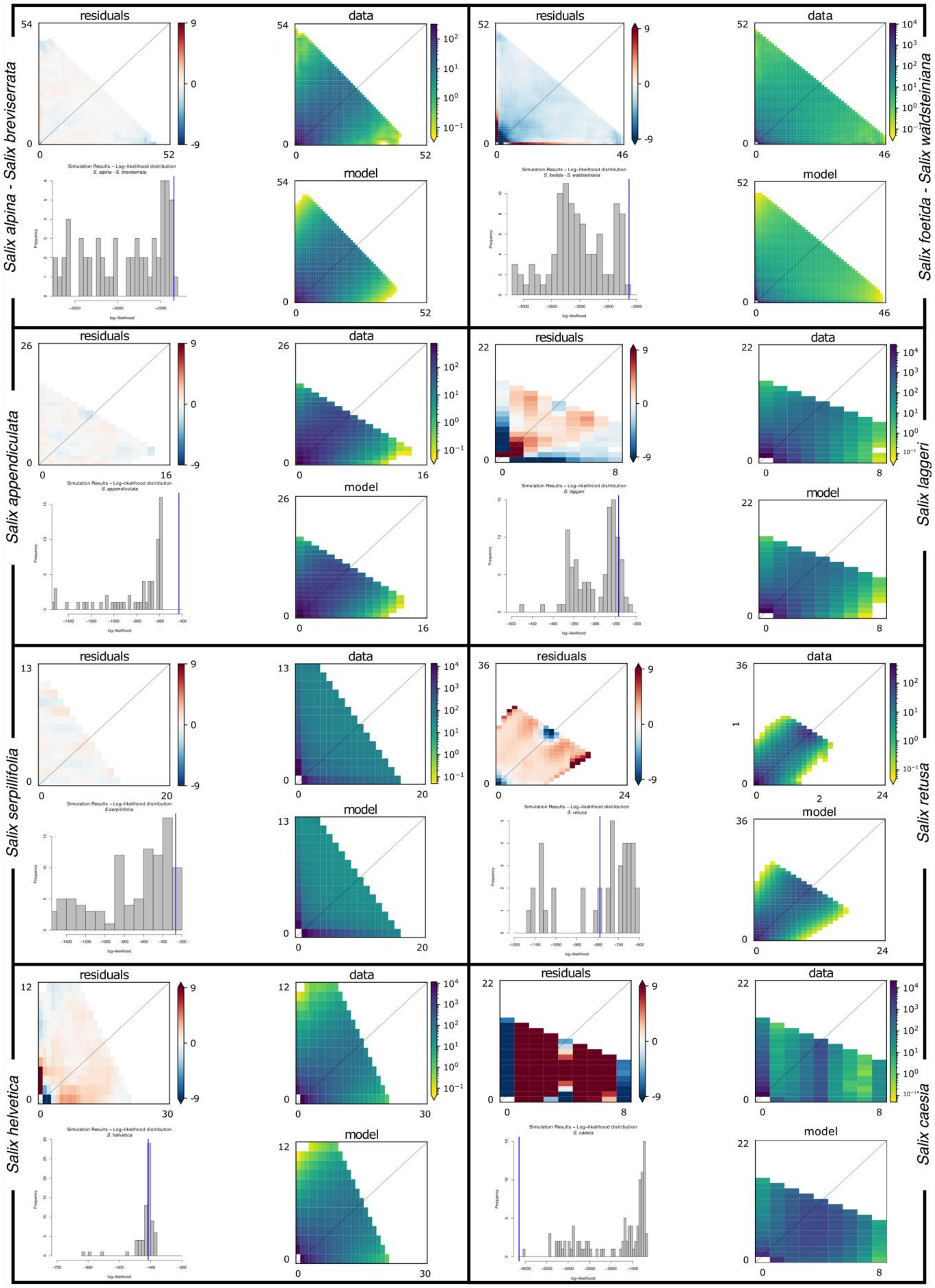

Figure S8: Evaluation of the best models for the diversification of the genetic groups (Figure 3). For the best model of each species, the empirical joint site frequency spectrum (top right), the joint site frequency spectrum inferred by the model (bottom right), the corresponding residuals (top left) between empirical and modeled data, and the estimated log-likelihood (blue line) and their distribution from the goodness-of-fit test (100 replicates) are displayed.

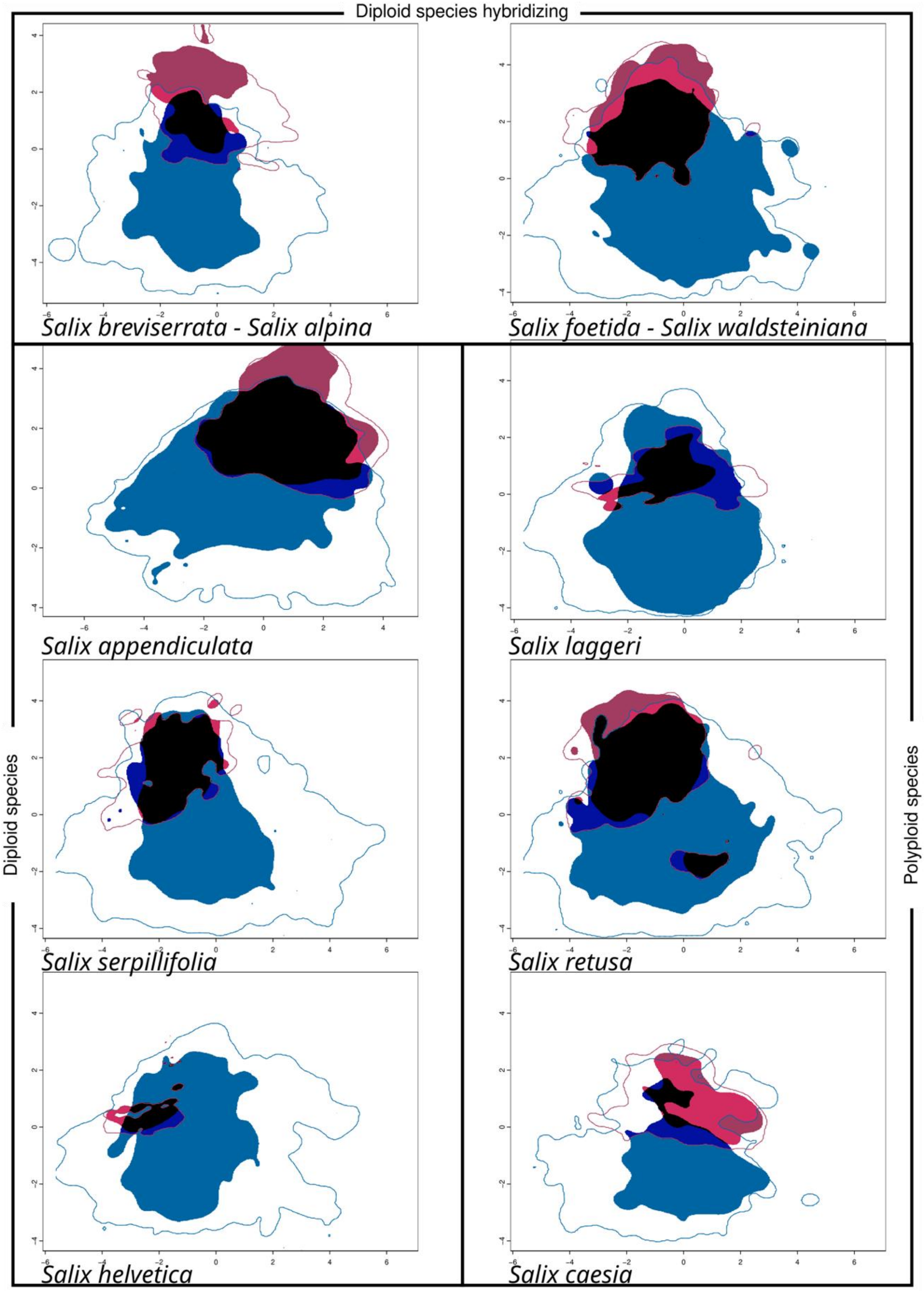

Figure S9: Summary of niche overlap between genetic groups or sister species pairs. Plots of niche overlaps in the two-dimensional space obtained by principal component analysis from the soil and bioclimatic variables. Niche stability is shown in black, niche expansion is shown by the group, respectively species, specific colours, i.e. blue for the western group and red for the eastern group. Solid outlines define the extent of the environmental conditions with each range.

Section S1: SNP filtering for different analyses.

STRUCTURE:
From the Stacks catalogs of diploid species, a single SNP per locus was selected to discard linked loci, and loci with excess heterozygosity (>60%) were removed to avoid calling of paralogous loci (--write-single-snp and –max-obs-het flags in *populations.pl*). Using PLINK v.1.90 [6], individuals with more than 15% missing genotype rate and sites with more than 10% missing data were removed. From the Stacks catalog of polyploid species, genotypes were called with the R package polyRAD [3], which takes the ploidy level into consideration. The package estimates genotype probabilities from read depth and structure in polyploids. Loci were filtered out if the percentage of individuals with one or more reads for a locus was lower than 60% and if the total number of individuals with reads for the minor allele was lower than 4. The non-Mendelian loci were further filtered using the Hind/HE distribution as described in [7]. An overview of the filtering criteria and the full data-processing workflow is presented in Table **S1**.

Demographic modelling:
VCF files for both diploids and polyploids were exported from the Stacks catalogs using the *populations.pl*. As Stacks is designed for diploid data, the initial SNP discovery and dataset generation for all species relied on diploid assumptions. A single SNP per locus was kept, only SNPs with sequence data for at least 60% of the individuals (--write-single-snp and -R flags in *populations.pl*). Depth filters were applied with VCFtools to keep SNPs with mean coverage between 10 and 50 (--minDP 10, --maxDP 50), and genotypes with a minimum quality of 20 (--minGQ 20), following thresholds used in previous studies employing the same approach [5, 8-10]. We acknowledge that the reliance on diploid assumptions may contribute to poorer model fits and stronger residual deviations in polyploid species, reflecting current bioinformatic limitations in fully accommodating higher ploidy levels. An overview of the filtering criteria and the full data-processing workflow is presented in Table **S1**.

Section S2: Methodology description for goodness-of-fit tests.

For each species, we refitted the best-supported model using the optimized parameter estimates and scaled the resulting model spectrum by the inferred theta. Based on this fitted model, we generated 100 Poisson-resampled spectra to simulate datasets expected under the model. Each simulated spectrum was then re-optimized to obtain distributions of log-likelihood values and Pearson's chi-square statistics. We assessed model fit by comparing the statistics calculated from the observed data to these simulated distributions. If the empirical values fell outside the simulated ranges, we interpreted this as evidence that the selected model did not adequately capture the data and therefore did not reliably estimate parameters [11].

Section S3: Explanations for generation time estimates.

The generation time of 30 years is an estimate of a grand mean, supported by the generally slow growth and high age of alpine plants [12]. Actual onset of the first flowering period of a comparable subarctic dwarf shrub varied dramatically between individuals, depending on shoot size, nutrient stage, and age [13].

Screenings of willows in 1984 on the recently colonized subalpine glacier forefield of the Rhone glacier reported e.g. adult *S. helvetica* and *S. purpurea*, but not their hybrid [14]. A re-sampling of these populations in 2010-2012 by E.H. detected the newly formed hybrid of these two species; these adult shrubs (with catkins) were genetically confirmed to be an F1 generation [15]. These observations support a generation time of 25-30 years in the subalpine zone and might be even longer in the alpine zone.

Section S4: GBIF data sources

Salix alpina:

GBIF.org (21 October 2021) GBIF Occurrence Download https://doi.org/10.15468/dl.etgc6q

Salix appendiculata:

GBIF.org (21 October 2021) GBIF Occurrence Download https://doi.org/10.15468/dl.u7fcmy

Salix breviserrata:

GBIF.org (21 October 2021) GBIF Occurrence Download https://doi.org/10.15468/dl.japsec

Salix caesia:

GBIF.org (21 October 2021) GBIF Occurrence Download https://doi.org/10.15468/dl.7c2m4p

Salix foetida:

GBIF.org (21 October 2021) GBIF Occurrence Download https://doi.org/10.15468/dl.ycdp2q

Salix helvetica:

GBIF.org (21 October 2021) GBIF Occurrence Download https://doi.org/10.15468/dl.b5mvqz

Salix laggeri:

GBIF.org (21 October 2021) GBIF Occurrence Download https://doi.org/10.15468/dl.fjxrqh

Salix retusa:

GBIF.org (1 August 2024) GBIF Occurrence Download https://doi.org/10.15468/dl.e563ap

Salix serpillifolia:

GBIF.org (1 August 2024) GBIF Occurrence Download https://doi.org/10.15468/dl.k4yx3d

Salix waldsteiniana:

GBIF.org (21 October 2021) GBIF Occurrence Download https://doi.org/10.15468/dl.2ekux6

Section S5: Methodology description of occurrences data extraction and filtering.

For all species, occurrence data were compiled from multiple sources, each of which was subjected to specific filtering procedures to ensure data quality.

EVA Database:
Vegetation relevés containing the target species were extracted from three EVA projects (IDs: 143, 186, and 237). The data were filtered to remove duplicate entries, records dated prior to 1980, observations with coordinate precision of fewer than four decimals, and records with spatial uncertainties exceeding 10 meters.

InfoFlora:
Raw occurrence data were obtained from InfoFlora. Filtering criteria included the removal of absence records, observations with uncertain taxonomic identification, introduced populations, imprecise geographic data (uncertainty exceeding 10 m), and records dated before 1980.

Biodiv'Aura and Silene
Raw occurrence data were obtained from Silene Expert (https://expert.silene.eu/) and biodiv'Aura Expert (https://donnees.biodiversite-auvergne-rhone-alpes.fr). Filtering criteria included the removal of imprecise geographic data (uncertainty exceeding 10 m), and records dated before 1980

GBIF:
Occurrence data were also retrieved from the Global Biodiversity Information Facility (GBIF), following a modified version of the protocol described by [16]. We filtered data based on the "basis of record," retaining only entries classified as human observations, literature, material samples, general observations, and preserved specimens. Further filtering excluded duplicate records, entries prior to 1980, coordinates with fewer than four decimals, and spatial uncertainty exceeding 10 meters.

Personal Observations:
In addition to public databases, we incorporated personal field observations and herbarium specimen collected over the past 20 years.

The full, combined dataset was then plotted and visually inspected, and records considered spatially implausible were manually removed.

References

1. Catchen, J., et al., *Stacks: an analysis tool set for population genomics.* Mol Ecol, 2013. **22**(11): p. 3124-40.
2. Eaton, D.A.R. and I. Overcast, *ipyrad: Interactive assembly and analysis of RADseq datasets.* Bioinformatics, 2020. **36**(8): p. 2592-2594.
3. Clark, L.V., A.E. Lipka, and E.J. Sacks, *polyRAD: Genotype Calling with Uncertainty from Sequencing Data in Polyploids and Diploids.* G3: Genes, Genomes, Genetics, 2019. **9**(3): p. 663-673.
4. Portik, D.M., et al., *Evaluating mechanisms of diversification in a Guineo-Congolian tropical forest frog using demographic model selection.* Molecular Ecology, 2017. **26**(19): p. 5245-5263.
5. Záveská, E., et al., *Evidence for Glacial Refugia of the Forest Understorey Species Helleborus niger (Ranunculaceae) in the Southern as Well as in the Northern Limestone Alps.* Front Plant Sci, 2021. **12**: p. 683043.
6. Purcell, S., et al., *PLINK: a tool set for whole-genome association and population-based linkage analyses.* Am J Hum Genet, 2007. **81**(3): p. 559-75.
7. Clark, L.V., et al., *A population-level statistic for assessing Mendelian behavior of genotyping-by-sequencing data from highly duplicated genomes.* BMC bioinformatics, 2022. **23**(101): p. 101.
8. Rota, F., et al., *Survival in nunatak and peripheral glacial refugia of three alpine plant species is partly predicted by altitudinal segregation.* Molecular Ecology, 2024. **33**(9): p. e17343.
9. Carnicero, P., et al., *Postglacial range expansion of high-elevation plants is restricted by dispersal ability and habitat specialization.* Journal of Biogeography, 2022. **49**(10): p. 1739-1752.
10. Voisin, C., et al., *Spatiotemporal diversification of forest understorey species reveals the existence of multiple Pleistocene forest refugia in Central Europe.* bioRxiv, 2025: p. 2025.05.13.653645.
11. Barratt, C.D., et al., *Vanishing refuge? Testing the forest refuge hypothesis in coastal East Africa using genome-wide sequence data for seven amphibians.* Molecular Ecology, 2018. **27**(21): p. 4289-4308.
12. Körner, C., *Alpine Plant Life: Functional Plant Ecology of High Mountain Ecosystems*. 2021, Springer.
13. Karlsson, P.S. and A. Jacobson, *Onset of reproduction in Rhododendron lapponicum shoots: the effect of shoot size, age, and nutrient status at two subarctic sites.* Oikos, 2001. **94**(2): p. 279-286.
14. Lautenschlager-Fleury, D. and E. Lautenschlager-Fleury, *Der gletschboden, ein weidenparadies*. 1985: Basler Botanische Gesellschaft.
15. Gramlich, S., et al., *Evolution in situ: hybrid origin and establishment of willows (Salix L.) on alpine glacier forefields.* Heredity, 2016. **116**(6): p. 531-541.
16. Chauvier, Y., et al., *Influence of climate, soil, and land cover on plant species distribution in the European Alps.* Ecological Monographs, 2021. **91**(2): p. e01433.
